# Long-term patterns of body mass and stature evolution within the hominin lineage

**DOI:** 10.1098/rsos.171339

**Published:** 2017-11-08

**Authors:** Manuel Will, Adrián Pablos, Jay T. Stock

**Affiliations:** 1 Gonville and Caius College, University of Cambridge, Cambridge CB2 3QG, UK; 2 PAVE Research Group, Department of Archaeology, University of Cambridge, Cambridge CB2 3QG, UK; 3 Department of Early Prehistory and Quaternary Ecology, University of Tübingen, Schloss Hohentübingen, 72070 Tübingen, Germany; 4 Centro Nacional de Investigación sobre la Evolución Humana (CENIEH), Paseo Sierra de Atapuerca 3, 09002 Burgos, Spain; 5 Grupo de Bioacústica Evolutiva y Paleoantropolgía (BEP), Área de Antropología Física, Departamento de Ciencias de la Vida, Universidad de Alcalá, Alcalá de Henares, 28871 Madrid, Spain; 6 Centro Mixto UCM-ISCIII de Investigación sobre Evolución y Comportamiento Humanos, c/Monforte de Lemos, 5, 28029 Madrid, Spain; 7 Department of Anthropology, Western University, London, Ontario, Canada N6A 3K7

**Keywords:** human evolution, body size, palaeoanthropology, *Homo*, *Australopithecus*

## Abstract

Body size is a central determinant of a species' biology and adaptive strategy, but the number of reliable estimates of hominin body mass and stature have been insufficient to determine long-term patterns and subtle interactions in these size components within our lineage. Here, we analyse 254 body mass and 204 stature estimates from a total of 311 hominin specimens dating from 4.4 Ma to the Holocene using multi-level chronological and taxonomic analytical categories. The results demonstrate complex temporal patterns of body size variation with phases of relative stasis intermitted by periods of rapid increases. The observed trajectories could result from punctuated increases at speciation events, but also differential proliferation of large-bodied taxa or the extinction of small-bodied populations. Combined taxonomic and temporal analyses show that in relation to australopithecines, early *Homo* is characterized by significantly larger average body mass and stature but retains considerable diversity, including small body sizes. Within later *Homo*, stature and body mass evolution follow different trajectories: average modern stature is maintained from *ca* 1.6 Ma, while consistently higher body masses are not established until the Middle Pleistocene at *ca* 0.5–0.4 Ma, likely caused by directional selection related to colonizing higher latitudes. Selection against small-bodied individuals (less than 40 kg; less than 140 cm) after 1.4 Ma is associated with a decrease in relative size variability in later *Homo* species compared with earlier *Homo* and australopithecines. The isolated small-bodied individuals of *Homo naledi* (*ca* 0.3 Ma) and *Homo floresiensis* (*ca* 100–60 ka) constitute important exceptions to these general patterns, adding further layers of complexity to the evolution of body size within the genus *Homo*. At the end of the Late Pleistocene and Holocene, body size in *Homo sapiens* declines on average, but also extends to lower limits not seen in comparable frequency since early *Homo*.

## 1. Introduction

Body size is one of the most important determinants of the biology of a species, as it correlates with metabolic rate, life history, energetic expenditure, diet, thermoregulation and home range size [1–3]. Palaeoanthropologists have estimated the body size of many hominin genera and species [4–10], but renewed interest in body size and shape has now provided novel data on diverse temporal and taxonomic parts of hominin history such as Middle and Late Pleistocene *Homo* [11–14], *Homo erectus/ergaster* [15–17], early *Homo* [18–20], *Homo* in general [15,21,22], earlier hominins before 1.5 Ma [23,24] and individual fossils such as KNM-WT 15000 [25]. While these studies provide deeper insights into particular taxa or temporal contexts, there has been no systematic and long-term overview of the evolution of body size within the hominin lineage. Recent improvements in the resolution of data now allow for broad comparisons of the evolution of body size throughout the last 4.4 Myr of hominin history with greater resolution than the landmark studies by McHenry [8] and Ruff *et al.* [10].

Such a long-term and inter-taxonomic analysis is important for several reasons. First, body size is central to discussions of human evolution throughout its entire temporal and geographical span [22,24]. Size is relevant to understanding the origin, taxonomy and adaptive strategies of *Homo*, *Australopithecus* and *Paranthropus* [11,21,23,24,26–31], energetics, locomotor adaptations and the first hominin dispersals into Eurasia [16,17,20,28,32–36] as well as rates of maturation and life-history parameters [37,38]. Body size estimates are also required as baseline for the comparative study of encephalization and brain evolution [39–41]. Recent studies have yielded novel developments in methodology and their applications to hominin fossils that provide ever-more and more accurate body size estimates [14,16,20,23,25,42–46].

Despite the range of recent research into the evolution of hominin body size, spatially or temporally restricted studies cannot address questions on larger scales. Although there is evidence for a temporal increase in body size throughout human evolution [4,6,8,28,30] (but see [23,24]), small sample sizes have restricted the application of statistical tests and little is known about the pattern and timing of these changes, even from a strict chronological perspective. Recent approaches have neglected to examine stature in favour of body mass, with no study analysing the subtle interactions of both size components. Some studies have, however, focused on the evolution of body shape, particularly on inter-limb proportions and the relation of hind-limb length to body mass [21,24,28,47].

The widely accepted interpretation of a major shift in body size with the origin of *Homo ergaster/erectus* (at around 1.8 Ma) in relation to *Homo habilis* and *Homo rudolfensis* has come under criticism [20], as has the notion of a marked size increase at the origin of *Homo* compared with australopithecines [23,24]. Recent analyses have also demonstrated that body size within early *Homo* is more spatially and temporally variable than previously acknowledged [20,30,46]. The scope of these studies, however, did not allow the contextualization of variability in body size throughout the Plio-Pleistocene. Temporal analyses during later periods of human evolution suggest an increase in body size during the Middle and Late Pleistocene [11,13], but these studies did not include large comparative samples of hominins greater than 1.0 Ma. The most recent body size estimates for *Homo naledi* [48]—dated to a surprisingly young age of *ca* 335–236 ka [49]—have also not been part of recent larger-scale comparative studies of body size that where published before these findings [20,23,24].

Several methodical problems and limitations impede the study of body size from a long-term and inter-taxonomic perspective. Most studies concerning hominin body size have provided species-means [8,10,23] which are often based on small numbers of fossils (*n* < 5) and unreliable attributions of alpha taxonomy (see discussion in [20,23]). While these studies are tailored to their specific research question and provide individual estimates of high accuracy, such an approach has led to a neglect of the amount and importance of variability, with the extent of intra- and interspecies variation in body size remaining largely unknown. This becomes all the more important as phenotypic variability—discussed under concepts such as variability selection [50] and phenotypic plasticity [30,34,51,52]—has recently become a common point of discussion with regards to the origins and evolution of our genus.

The prevalence of small sample sizes also more generally affects the study of long-term and inter-taxonomic patterns in hominin body size evolution and the application of statistical methods. For groups with very small sample sizes (*n* < 5), the effect of outliers—which might result from individual prediction errors, unreliable estimation methods, incorrect taxonomic attributions or chronological ages—on central tendencies and measures of variation is particularly strong (for discussion in palaeoanthropology, see [53–55]). Even when estimates for individual specimens are accurate, means of such groups with small sample sizes are unlikely to adequately estimate original population means and might thus not be representative for past demes. The low signal-to-noise ratio for such samples can only be enhanced by increasing sample sizes.

The importance of sample sizes to the detection of statistical trends has been known since John Graunt's ‘Natural and Political Observations Made Upon the Bills of Mortality’ in 1662. According to the principle of statistical regularity, an umbrella term that also encompasses the law of large numbers, statistical regularities emerge when sample size increases, meaning that random and rare variation (or error) has less weight on overall patterns. Using large sample sizes also aids in finding patterns that are not detectable using restricted samples. From a statistical point of view, standard parametric methods are not applicable to small samples, and even non-parametric approaches are considered unreliable for analytical categories with *n* < 5 [56–58]. In sum, small sample sizes constitute the key limitation for large-scale studies across the whole timeframe of hominin evolution which necessitate as large samples as possible to increase the signal-to-noise ratio and examine patterns of body size evolution in appropriate detail at various levels.

## 2. Material and methods

### 2.1. Approach and research questions

In order to assess long-term and inter-taxonomic patterns of body mass and stature evolution within the hominin lineage, the general approach of our study was to tackle the essential problem of small sample sizes for hominin body size estimates by gathering as many reliable predictions as possible from our own work and published sources. By compiling a large database on body size of hominins (*n* = 311) from three continents and employing multi-tiered chronological and taxonomic analytical categories, we systematically assess the temporal and taxonomic evolution of body size throughout the last 4.4 Myr regarding both body mass and stature. Importantly, the large sample allows the application of statistical methods to test for inter-group differences.

We characterize change in both size and variability through human evolution and assess more specific hypotheses divided into temporal (1) and taxonomic (2) questions: (1) Did hominin body size increase in a more gradual or punctual manner throughout the past 4.4 million years? Did body mass and stature evolve in concert or follow separate trajectories? Does variability in size change through time? (2) Was the origin of *Homo* characterized by higher variability or larger body size in comparison to *Australopithecus* and *Paranthropus*? Are there differences in body mass, stature and shape among more recent *Homo* species?

### 2.2. Collection of data

We combined our own estimates of body mass and stature among early *Homo* between 2.2 and 1.4 Ma [20] with new body size estimates for *Homo* from tarsal bones throughout the entire Pleistocene (e.g. [14]) and the latest published data from other recent key studies that pre- and postdate this time frame (electronic supplementary material, file S1). For body mass estimates greater than 2.2 Ma, we used mainly data by Grabowski *et al.* [23] as their methods were specifically designed for predicting smaller-bodied australopithecines, and additionally collected (stature) estimates for specimens from McHenry [7,8]. The period after 1.4 Ma is represented by new estimates on tarsal bones (by A.P. [14,59,60]) with additional data from larger comparative analyses (e.g. [10,11,13]). The body size estimates are provided by specimen in electronic supplementary material, file S1, together with their source, chronological and taxonomic information, methods used to estimate body size and prediction errors (95% confidence intervals) [61]. Selection criteria for estimates in the Sima de los Huesos palaeo-population are provided in electronic supplementary material, text S1 and file S2.

In total, our sample encompasses size estimates of 311 hominin fossils of several species and genera (*Homo*: *n* = 241; *Australopithecus*: *n* = 48; *Paranthropus*: *n* = 21; *Ardipithecus*: *n* = 1), with *n* = 254 estimates of body mass and *n* = 204 estimates of stature (electronic supplementary material, file S1). The fossil hominin dataset covers roughly 4.4 Myr from the Late Pleistocene to the end of the Pleistocene (4.4–0.011 Ma) from Africa, Europe and Asia. In order to contextualize results with more recent time periods, we incorporated body size estimates of a global hunter–gatherer sample (*n* = 828) from *ca* 10 ka until the recent past (‘Holocene foragers’ [20]).

Our approach of maximizing sample sizes of hominin body size estimates to detect large-scale patterns with statistical methods comes with the cost of incorporating individual body size estimates from different studies, estimation methods and skeletal elements which introduce errors. These methodical trade-offs are common in meta-studies and can be justified by the rationale and aims of the study (see above). While a complete removal of error sources is impossible, control of quality and commensurability of body size estimates was an important concern of the study design in order to minimize these problems as far as possible. We performed this task by assessing several of the previously raised points as well as other prominent issues in body size estimates [20,23]: (i) removing unreliable and aberrant values; (ii) comparing between different studies; (iii) comparing between different methodologies; (iv) controlling for body part representation; and (v) assessing fragmented specimens. Methods for these controls are described in the following in order to provide transparency and replicability of our approach. We also refer to the respective datasets and more detailed summary of results.

(i) Unreliable and aberrant values were removed when reported as such in the literature (e.g. estimates from old studies whose methods are not deemed adequate according to modern standards) and are not included in this study. (ii) Individual fossils for which estimates diverged strongly between different key studies (greater than 30% for body mass; greater than 20% for stature) were removed, and the remaining sample analysed separately (see electronic supplementary material, table S1). Results of this approach were checked for consistency against results deriving from the entire database, ensuring that they do not bias the overall analysis (see summary in electronic supplementary material, text S2). This approach was necessary due to large differences in body mass estimates for some early *Homo* fossils greater than 1.0 Ma between McHenry [8], Grabowski *et al.* [23] and Will & Stock [20], summarized in Jungers *et al.* [24]. (iii) We studied potential bias introduced by using estimates from different studies with diverging methodologies for selected temporal and taxonomic groups in which we found significant changes (see electronic supplementary material, text S3; file S3; tables S2 and S3). (iv) Body part representation was assessed to control for potential bias between different analytical categories, but found little effect on the overall results (see electronic supplementary material, table S4). (v) The fragmentary nature of some fossils included in our database means that the assessment of body size in these cases carries considerable error margins, as do all estimates of body size in fossil hominins to different degrees [8,10,11,16,20,23,61]. However, we decided to include them—trading data quality for quantity to a certain extent—in order to: (i) incorporate the current fossil record as available and remove bias of including only complete specimens which provide a potentially distorted picture; (ii) achieve the best possible resolution of chronological periods and taxonomic groups; and (iii) answer the specific questions set out above. As we have shown in a previous study [20] removing these estimates that make up a minority of the sample—such as OH 62 and KNM-ER 3735—has little effect on large-scale patterns of relative changes, measures of central tendency and dispersion, as well non-parametric statistical tests between groups of larger sample size. These trade-offs can be justified by the law of statistical regularity, whereby increasing sample sizes will also lead to a better differentiation between signal and noise in the data. Importantly, another advantage of this approach is that it allows for the application of statistical tests which most previous studies of predominantly small sample sizes were not able to do.

### 2.3. Analysis of data

Our analysis of estimates follows along two independent variables: age and taxonomic assignment. Chronometric ages were taken from the literature. We focus on temporal analyses as time is a more reliable variable than taxonomy. Body size was regressed on absolute time to assess general patterns and timing of change. We investigated chronological trends in more detail by dividing the hominin sample into discrete temporal groups of different resolution (‘fine’ and ‘coarse’). The groups are provided in electronic supplementary material, table S5, with the attribution of individual specimens to these groups found in electronic supplementary material, file S1. Means and coefficients of variation were compared between groups to identify potential periods of step increases or gradual change. Since phylogenetic relationships between most hominin species are highly controversial [20,23,26,27,29,37], we approximate evolutionary change by detailed continuous and discrete chronological analyses across lineages (see [40] for comparable approach for brain size evolution). This approach, however, introduces increased error in mean values for any given time period (i.e. temporal groups) for which multiple hominin species have coexisted. We thus also account for evolutionary rates within taxa, regressing absolute time by body size within our taxonomic categories to look for stasis or continuous change among these groups (see also [40]). In order to gain a better understanding of evolutionary rates within evolving lineages, a confident hominin phylogeny with clear ancestor–descendant relationships is required; however, there is currently no consensus. The application of a hypothetical phylogeny to the analyses thus risks the introduction of additional errors. Our ‘non-phylogenetic’ approach uses information from both taxonomy (as proxy for phylogenetic hierarchy) and time (earlier taxa are probable ancestors to later taxa) to approximate these relationships (for more detail on this issue, see [62]).

We took into account taxonomic designation of the hominin fossils, both to the level of the genus and the species where possible. There are important caveats with this approach such as the fragmentary and isolated nature of some postcranial fossils, the lack of consensus regarding many hypodygms, and multiple taxonomic attributions for individual fossils (see summaries in [20,23]). While a broad assignment to the genus level is considered to be more robust, the alpha taxonomy of some specimens—particularly for early *Homo* and generally hominin fossils between 2.5 and 1.5 Ma—is highly debated and often unreliable due to the above reasons. To circumvent these problems, we used both broad (higher confidence of attribution but low resolution) and narrow taxonomic groupings (lower confidence of attribution but higher resolution) as analytical categories, with a focus on the former which trades specificity for reliability (see [40] for comparable approach). The groups are provided in electronic supplementary material, table S6, with the attribution of individual specimens to these groups found in electronic supplementary material, file S1. The taxonomic information for these groupings was taken from the literature with the principle of majority rule applied. We also sought comparability to previous studies that estimated body mass with explicit taxonomic assessment and consideration of these problems [7,8,11,13,15,23,24].

To assess variation in body size in relationship to other independent variables, we performed statistical analyses on the Plio-Pleistocene hominin sample using SPSS 24.0. As one primary interest is the variability of body size, we calculated coefficients of variation as a relative measure of variability rather than absolute variability, as the latter increases with trait size [63]. Mean and median values were calculated for all groups based on reported point estimates for individual specimens. To account for prediction intervals around individual fossil estimates [61], we also calculated lower and upper confidence boundaries around the mean for each analytical group [23]. For comparing means between more than two groups we performed two-tailed ANOVA or Kruskal–Wallis tests to investigate significant differences (*p *< 0.05). *Post hoc* comparisons among more than two groups were made using Games–Howell or Mann–Whitney *U*-tests with Bonferroni's correction to protect against Type I errors.

## 3. Results

### 3.1. Variation of hominin body size by time

**Figure 1:**
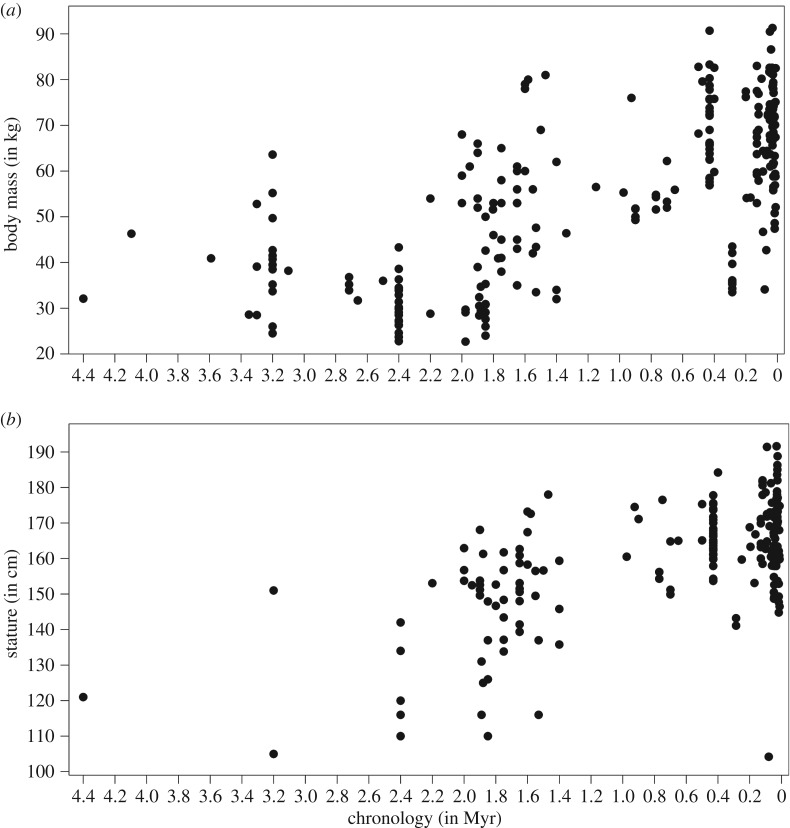
Body size estimates by time in the entire sample of fossil hominins. (*a*) Body mass (in kg); (*b*) stature (in cm).

Linear regressions of body size by chronometric age (figure 1) find a strong positive and highly significant correlation with body mass (*F* = 226.551; *p *< 0.0001; *R*^2^ = 0.475; d.f. = 250) and stature (*F* = 125.337; *p *< 0.0001; *R*^2^ = 0.384; d.f. = 201). Nonlinear line-fitting provides slightly higher *R*^2^-statistics but comparable or lower *F*- and *p*-values (electronic supplementary material, table S7). While these different models indicate a chronological trend of increasing body size through the past 4.4 Ma, they only account for approximately 40–50% of observed variation and cannot track the more complex variation evident in figure 1. The temporal pattern of generally increasing size is interrupted at two points: first, by a marked reduction in body mass estimates after 3.2 Ma followed by an increase from 2.2 Ma onwards until the Middle Pleistocene (*ca* 0.4 Ma); and second at *ca* 0.3 Ma, followed by another rise in body size values (see below; also electronic supplementary material, figures S1 and S2). The data distribution in figure 1 demonstrates abundant variability in body size estimates within most timeframes, but particularly between 2.0 and 1.4 Ma. Large body mass and tall stature (greater than 70 kg; greater than 170 cm) are occasionally reached by *ca* 1.6 Ma but are frequent only after 0.5 Ma, particularly for body mass. Conversely, small body sizes (less than 40 kg; less than 140 cm) predominate between 4.0 and 2.2 Ma and are virtually absent after 1.4 Ma, with notable exceptions at *ca* 0.3 Ma (*n* = 6; coinciding with *Homo naledi*) and at *ca* 0.08 Ma (*n* = 1; coinciding with *Homo floresiensis*).

**Figure 2.**
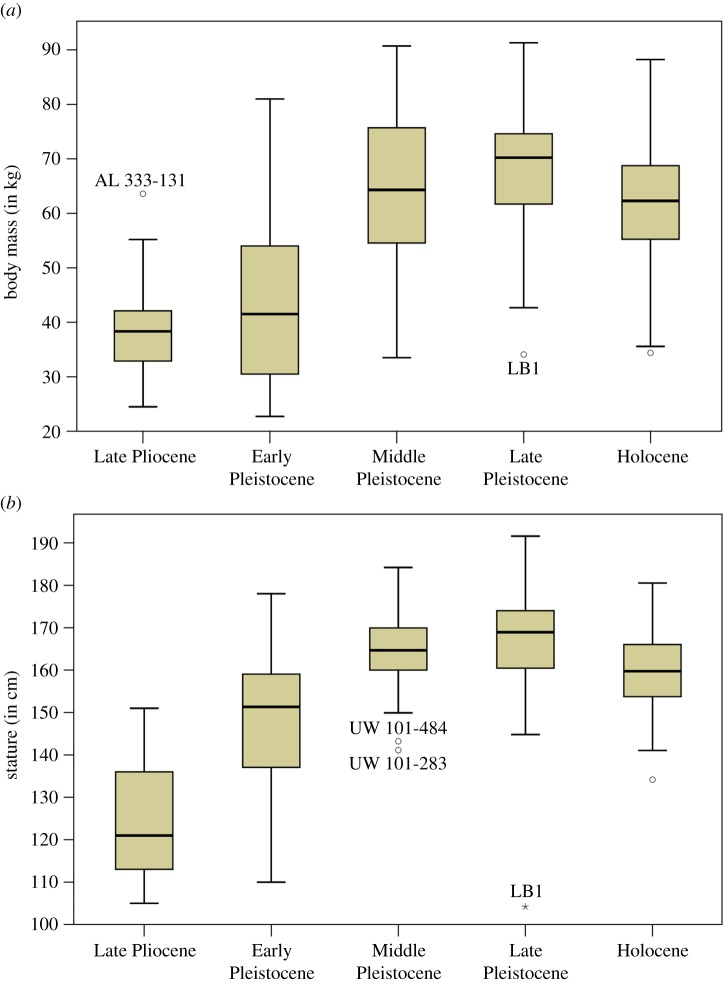
Body size estimates by coarse temporal groups. (*a*) Body mass (in kg) and (*b*) stature (in cm).

Comparing hominin samples partitioned by coarse temporal groups (electronic supplementary material, table S5) shows that both Middle and Late Pleistocene hominins feature large body masses (on average approx. 63–70 kg), with the Early Pleistocene and Late Pliocene exhibiting markedly lower mean values (less than 45 kg; table 1 and figure 2). ANOVA indicates that variation between groups is significant (*F*_3,248_ = 69.725; *p *< 0.001) with Games–Howell *post hoc* test finding significant differences between the Late Pleistocene/Middle Pleistocene versus Early Pleistocene/Late Pliocene (*p *< 0.001) each. Results are nearly identical for stature: hominins from the Middle and Late Pleistocene exhibit significantly larger average heights (approx. 164–169 cm) than during the Early Pleistocene (approx. 148–151 cm; table 1; ANOVA: *F*_2,197_ = 40.805; *p *< 0.001; Middle/Late Pleistocene versus Early Pleistocene Games–Howell: *p *< 0.001), with the difference that the Late Pliocene (mean: 125.7 cm) was removed from statistical analyses due to low sample size (*n* = 3).

**Table 1.** Results of the chronological analyses of hominin body size estimates by coarse temporal groups.

coarse temporal group	*n*	mean	median	mean CI L^a^	mean CI U^a^	s.d.	min	max	CV
body mass									
Holocene^b^	438	61.86	62.28	—	—	10.71	34.39	88.23	17.3
Late Pleistocene	78	68.69	70.20	54.95	82.43	10.69	34.10	91.30	15.6
Middle Pleistocene	60	63.28	64.30	52.13	75.64	13.93	33.50	90.70	22.0
Early Pleistocene	90	43.84	41.50	31.77	60.79	15.13	22.70	81.00	34.5
Late Pliocene	24	38.95	38.50	25.10	60.90	9.41	24.50	63.60	24.2
stature									
Holocene^b^	449	159.91	159.74	—	—	8.28	134.16	180.55	5.2
Late Pleistocene	92	167.14	168.95	153.78	180.52	12.24	104.20	191.60	7.3
Middle Pleistocene	52	164.39	164.65	156.40	172.40	8.48	141.10	184.20	5.2
Early Pleistocene	56	148.02	151.35	137.56	158.57	16.66	110.00	178.00	11.3
Late Pliocene	3	125.67	121.00	115.80	135.20	23.35	105.00	151.00	18.6

^a^Lower and upper 95% confidence intervals of the mean (individual prediction intervals in electronic supplementary material, file S1).

^b^The Holocene forager sample is provided as comparative baseline and excluded from the statistical analyses.

Variability of body mass within coarse time groups (table 1) is particularly high in the Early Pleistocene (CV = 34.5%), with lower values for the Late Pliocene (CV = 24.2%) and Middle Pleistocene (CV = 22.0%). Reduced levels of variation characterize the Late Pleistocene (CV = 15.6%) which is slightly below the Holocene sample (CV = 17.3%). Comparable results are found for stature, with peak variance during the Late Pliocene (CV = 18.6%) and Early Pleistocene (CV = 11.3%). Here, however, both the Middle and Late Pleistocene show low values (CV = 5.2–7.3%) close to the Holocene sample (CV = 5.2%).

**Figure 3.**
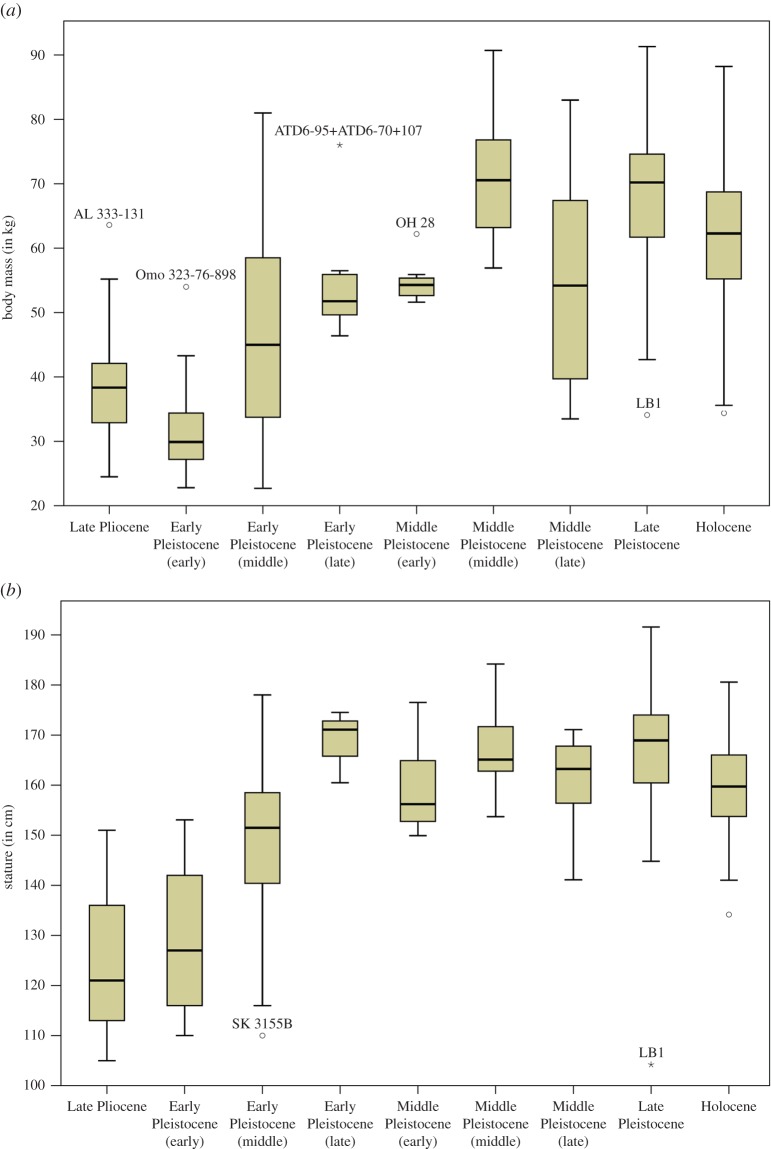
Body size estimates by fine temporal groups. (*a*) Body mass (in kg) and (*b*) stature (in cm).

When analysing temporal trends among hominins grouped into finer time slices (electronic supplementary material, table S5), summary statistics (table 2) and box plots (figure 3) indicate three coherent groups that underscore a general trend towards increasing size through time with some important nonlinear deviations. The lowest body mass values are found in the Late Pliocene and early Early Pleistocene (mean: 31.7–39.0 kg; median: 29.9–38.4 kg)—–with the former showing larger values compared with the latter—followed by the middle Early Pleistocene until the early Middle Pleistocene, but also including the late Middle Pleistocene (mean: 47.1–55.2 kg; median: 45.0–54.3 kg). The largest and youngest group encompasses the middle Middle Pleistocene as well as the Late Pleistocene (mean: 68.7–70.4 kg; median: 70.2–70.6 kg). The analyses find four marked temporal shifts in body mass between neighbouring time slices with a difference of around 15 kg on average. These include increases between the early Early Pleistocene versus middle Early Pleistocene, the early Middle Pleistocene versus middle Middle Pleistocene and the late Middle Pleistocene versus Late Pleistocene, as well as decreases between the middle and late Middle Pleistocene (caused entirely by the *Homo naledi* specimens in the latter group; see electronic supplementary material, figures S3 and S4). A Kruskal–Wallis test shows that overall differences between time slices are significant (*H*_7_ = 139.130; *n* = 252; *p *< 0.001), but only the two major increases between early Early Pleistocene versus middle Early Pleistocene (*p *= 0.030) and the early Middle Pleistocene versus middle Middle Pleistocene (*p *< 0.001) reach significance. Testing these findings by different methods (electronic supplementary material, text S3 and table S2) underscores the robustness of the marked and significant increases. Analyses of stature with the same age groupings provide similar results (table 2 and figure 3), but exhibit an even larger increase in height between the early and middle Early Pleistocene (approx. 20 cm on average). An important difference from the analysis of body mass is an earlier shift in stature between the middle and late Early Pleistocene (approx. 20 cm on average) after which height remains on comparably high average levels (approx. 160–169 cm). Statistical testing of these groupings is precluded by very small sample sizes for some groups (*n* = 3) in relation to overall group numbers (*n* = 8).

**Table 2.** Results of the chronological analyses of hominin body size estimates by fine temporal groups.

fine temporal group	*n*	mean	median	mean CI L^a^	mean CI U^a^	s.d.	min	max	CV
body mass									
Holocene^b^	438	61.86	62.28	—	—	10.71	34.39	88.23	17.3
Late Pleistocene	78	68.69	70.20	54.95	82.43	10.69	34.10	91.30	15.6
late Middle Pleistocene	21	55.24	54.20	49.90	60.60	16.36	33.50	83.00	29.6
middle Middle Pleistocene	32	70.40	70.55	56.51	84.55	9.05	56.90	90.70	12.9
early Middle Pleistocene	7	54.87	54.30	35.70	81.69	3.57	51.60	62.20	6.5
late Early Pleistocene	8	54.63	51.75	37.45	80.10	9.22	46.40	76.00	16.9
middle Early Pleistocene	59	47.11	45.00	35.24	62.94	15.42	22.70	81.00	32.7
early Early Pleistocene	23	31.73	29.90	20.89	48.56	6.96	22.80	54.00	21.9
Late Pliocene	24	38.96	38.35	25.10	60.89	9.42	24.50	63.60	24.2
stature									
Holocene^b^	449	159.91	159.74	—	—	8.28	134.16	180.55	5.2
Late Pleistocene	92	167.14	168.95	153.78	180.52	12.24	104.20	191.60	7.3
late Middle Pleistocene	12	160.37	163.25	153.43	167.58	9.86	153.10	171.10	6.1
middle Middle Pleistocene	33	166.85	165.10	158.92	174.75	6.85	153.70	184.20	4.1
early Middle Pleistocene	7	159.70	156.20	149.77	169.83	9.54	149.90	176.50	6.0
late Early Pleistocene	3	168.70	171.10	155.40	181.83	7.30	161.70	174.50	4.3
middle Early Pleistocene	47	149.11	151.49	138.85	159.45	14.97	110.00	178.00	10.0
early Early Pleistocene	6	129.18	127.00	118.76	141.00	16.63	110.00	153.05	12.9
Late Pliocene	3	125.67	121.00	115.80	135.20	23.35	105.00	151.00	18.6

^a^Lower and upper 95% confidence intervals of the mean (individual prediction intervals in electronic supplementary material, file S1).

^b^The Holocene forager sample is provided as comparative baseline and excluded from the statistical analyses.

Plotting the chronological group boundaries on scatter plots of body size estimates (electronic supplementary material, figures S5 and S6) shows that these more pronounced shifts are not merely an artefact of specific groupings that masks a more continuous increase, but derive largely from the data structure. An even finer resolution of analysis—calculating the mean of body size estimates by 100 ky increments—supports the more punctuated than gradual manner of changes throughout time (electronic supplementary material, figures S1 and S2), but is limited by small sample sizes for some 100 ky brackets.

Body mass and stature (table 2) are particularly variable from the Late Pliocene to the middle Early Pleistocene (CV mass = 21.9–32.7%; CV stature = 10.0–18.6%), but also for the late Middle Pleistocene for body mass only (CV = 29.6%). Reduced levels of variation without overlap to these temporal categories characterize the late Early Pleistocene to Late Pleistocene (range of CVs: body mass = 6.5–16.9%; stature = 3.3–7.3%) and stature in the late Middle Pleistocene (CV = 6.1%). These values lie close to or even below those of our recent global sample of hunters and gatherers (CV body mass = 17.3%; CV stature = 5.2%). CVs of both temporal analyses suggest a relatively consistent decrease in body size variability through time—except for body mass in the late Middle Pleistocene with the inclusion of small-bodied *Homo naledi* (see below)—with the most recent time slices exhibiting CVs comparable to or slightly below the Holocene sample.

### 3.2. Variation of hominin body size by taxonomic category

**Figure 4.**
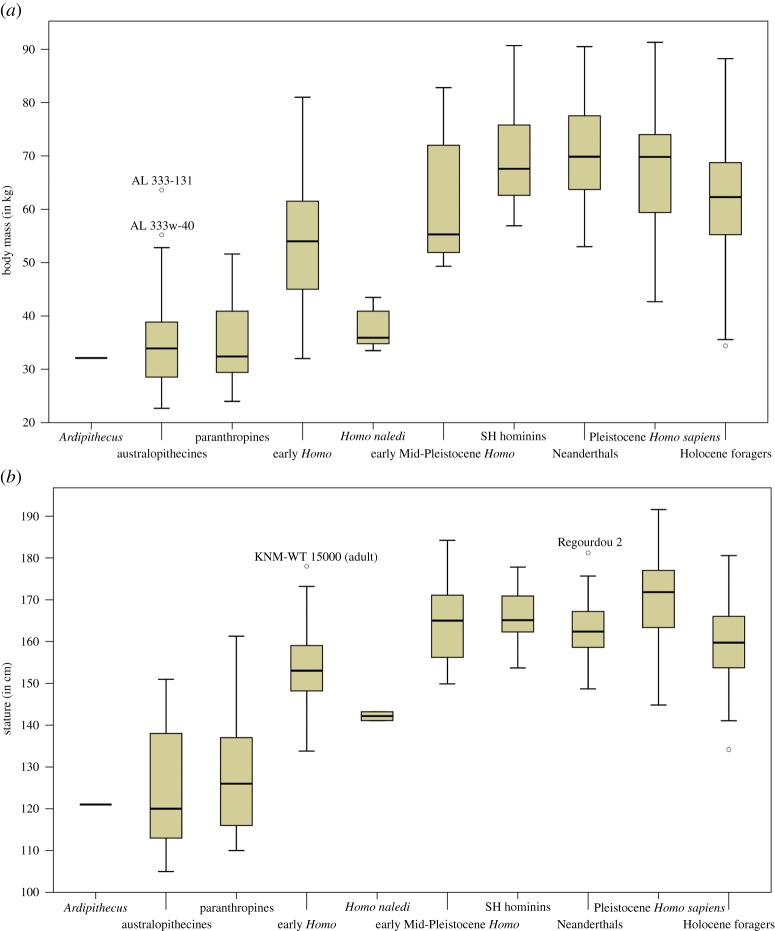
Body size estimates by broad taxonomic groupings. (*a*) Body mass (in kg) and (*b*) stature (in cm).

Variation of hominin size estimates divided by broad taxonomic groupings (electronic supplementary material, table S6) is illustrated in figure 4. Matching with the fine chronological analyses of increasing body mass, three groupings can be discerned (table 3). An early group with the lowest body mass including *Ardipithecus*, *Australopithecus* and *Paranthropus* (mean: 32.1–35.0 kg; median: 32.1–33.9 kg) but also *Homo naledi* from the late Middle Pleistocene (mean: 37.5 kg; median: 35.9 kg), followed by early *Homo* and early Mid-Pleistocene *Homo* (mean: 54.7–61.5 kg; median: 54.0–55.3 kg), and finally, the youngest and largest group encompassing the Sima de los Huesos (SH) hominins, Neanderthals, and Pleistocene *Homo sapiens* (mean: 67.2–70.5 kg; median: 67.6–69.9 kg). Combined with chronological considerations, the two major shifts in average body mass occur between *Australopithecus/Paranthropus* versus early *Homo* (approx. 20 kg) and between early Mid-Pleistocene *Homo* versus the SH hominins (approx. 10–15 kg). Overall differences between taxonomic groups are significant (*H*_8_ = 163.030; *n* = 249; *p* < 0.001), with *post hoc* comparisons revealing significant differences between *Australopithecus*/*Paranthropus* versus all other *Homo* groups (*p* < 0.003) except *Homo naledi* (*p *= 1.000), as well as between early *Homo* and *Homo naledi* versus SH hominins/Neanderthals/Pleistocene *Homo sapiens* (*p* < 0.019). Differences between early *Homo* and *Homo naledi* are not significantly different in this analysis.

**Table 3.** Results of the taxonomic analyses of hominin body size estimates by broad groupings, ordered through time from younger to older.

taxonomic grouping	*n*	mean	median	mean CI L^c^	mean CI U^c^	s.d.	min	max	CV
body mass									
Holocene foragers^a^	438	61.86	62.28	—	—	10.71	34.39	88.23	17.3
Pleistocene *Homo sapiens*^b^	57	67.16	69.80	53.72	80.58	10.16	42.70	91.30	15.1
Neanderthals	30	70.52	69.85	56.42	84.63	9.14	53.00	90.50	13.0
SH hominins	26	69.38	67.60	55.50	83.26	8.90	56.90	90.70	12.8
early Mid-Pleistocene *Homo*	19	61.49	55.30	44.35	84.74	12.08	47.10	82.60	19.6
*Homo naledi*	8	37.53	35.90	30.02	45.03	3.75	33.50	43.50	10.0
early *Homo*	40	54.73	54.00	42.68	67.41	12.84	32.00	81.00	23.5
paranthropines	21	35.04	32.40	21.41	56.92	7.70	24.00	51.60	22.0
australopithecines	47	34.64	33.90	22.99	54.23	8.79	22.70	63.60	25.4
*Ardipithecus*	1	32.10	32.10	19.40	53.30	—	—	—	—
stature									
Holocene foragers^a^	449	159.91	159.74	—	—	8.28	134.16	180.55	5.2
Pleistocene *Homo sapiens*^b^	60	170.34	171.85	153.78	184.62	10.64	144.80	191.60	6.2
Neanderthals	38	162.66	162.40	152.05	173.24	7.25	148.70	181.20	4.5
SH hominins	30	166.05	165.10	158.42	173.61	6.18	153.70	177.80	3.7
early Mid-Pleistocene *Homo*	13	164.84	165.00	153.92	175.95	10.36	149.90	184.20	6.3
*Homo naledi*	2	142.15	142.15	134.40	151.75	1.48	141.10	143.20	1.0
early *Homo*	39	153.89	153.05	144.45	164.00	10.04	133.82	178.00	6.5
paranthropines	9	128.81	126.00	112.28	137.23	15.41	110.00	161.30	12.0
australopithecines	7	125.43	120.00	117.87	140.95	17.20	105.00	151.00	13.7
*Ardipithecus*	1	121.00	121.00	117.00	124.00	—	—	—	—

^a^The Holocene forager sample is provided as comparative baseline and excluded from the statistical analyses.

^b^Combined data from Middle Palaeolithic (MP) *Homo sapiens* and Upper Palaeolithic modern humans. The Holocene forager sample is displayed separately.

^c^Lower and upper 95% confidence intervals of the mean (individual prediction intervals in electronic supplementary material, file S1).

Taxonomic analyses of stature find a first marked increase in early *Homo* compared with *Australopithecus* and *Paranthropus* (approx. 20–30 cm), concurrent with a shift to larger body masses (table 3 and figure 4). The second larger increase in mean stature is temporally decoupled from body mass, taking place earlier between early *Homo* and early Mid-Pleistocene *Homo* (approx. 10 cm), after which mean statures range between 162.7 and 170.3 cm with the exception of the *Homo naledi* specimens. *Homo naledi* is over 20 cm smaller than the temporally closest taxonomic group (i.e. SH hominins) and approximately 10 cm smaller compared with early *Homo*. Overall differences between the groups are significant (*H*_8_ = 90.001; *n* = 199; *p *< 0.001) and *post hoc* tests find significant differences between *Australopithecus*/*Paranthropus* versus all other *Homo* groups (*p *< 0.009)—except early *Homo* and *Homo naledi—*as well as between early *Homo* versus the SH hominins and Pleistocene *Homo sapiens* (*p *< 0.002). The analysis also identifies significantly lower stature among Neanderthals compared with Pleistocene *Homo sapiens* (*p *= 0.034).

Variation of body mass among broad taxonomic groupings (table 3 and figure 4) shows a separation between high levels of relative variability in *Australopithecus* (CV = 25.4%), *Paranthropus* (CV = 22.0%), early *Homo* (CV = 23.5%) and early Mid-Pleistocene *Homo* (CV = 19.6%), compared with reduced levels within the later *Homo naledi*, SH hominins, Neanderthals and Pleistocene *Homo sapiens* (CV range = 12.8–15.1%). For stature, *Australopithecus* (CV = 13.7%) and *Paranthropus* (CV = 12.0%) likewise exhibit high variation, followed by generally reduced levels for all groupings of *Homo* (CV range = 1.0–6.5%), matching well with CVs for the recent hunter–gatherer sample (CV = 5.2%).

**Figure 5.**
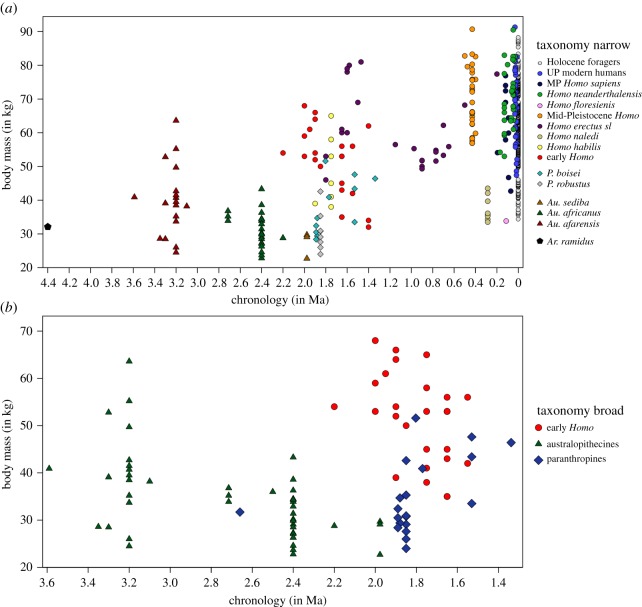
Body mass by time with indication of taxonomic group membership. (*a*) All fossil hominins by narrow taxonomy plus Holocene foragers and (*b*) zoom in the period 3.6–1.4 Ma to illustrate the difference in body mass around the origin of *Homo* (only early *Homo* specimens greater than 1.5 Ma not assigned to *Homo erectus s.l*. are shown) compared to *Australopithecus* and *Paranthropus* estimates.

**Figure 6.**
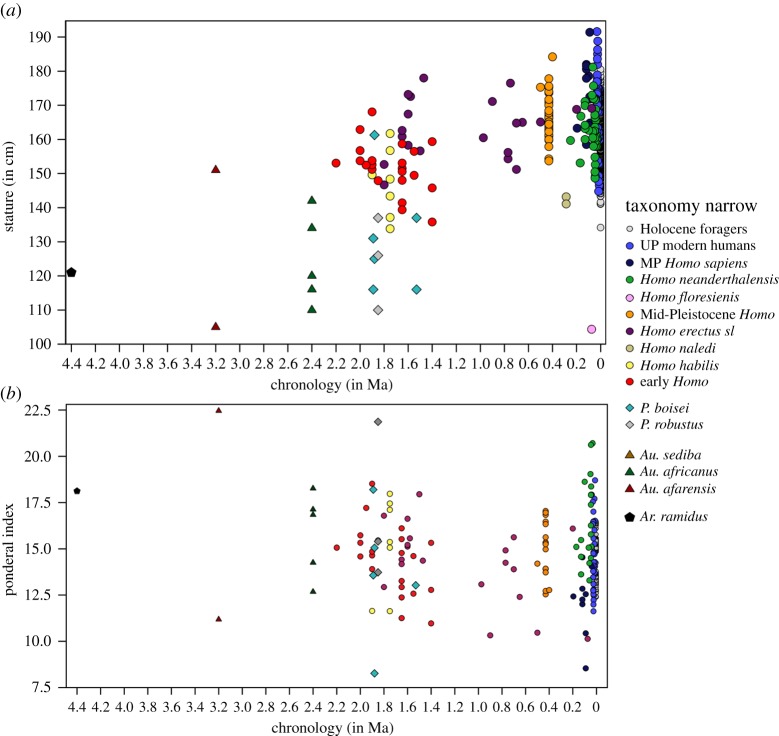
Stature and ponderal index by time for fossil hominins and Holocene foragers. (*a*) Stature estimates coded by narrow taxonomy and (*b*) Ponderal index values coded by narrow taxonomy. Higher values indicate relatively stockier builds.

A separate analysis assessed body size around the origin of *Homo*. The early *Homo* sample for this analysis includes all specimens between 2.2 and 1.6 Ma that are not assigned to *Homo erectus/ergaster.* This approach excludes large-bodied forms of *Homo* after 1.6 Ma (e.g. KNM-WT 15000; KNM-ER 736) as they were previously found to show significant increase in body size relative to earlier *Homo* fossils [20]. Statistical comparisons identify significant differences among early *Homo*, *Australopithecus* and *Paranthropus* regarding both body mass (ANOVA: *F*_2,94_ = 30.437; *p *< 0.001) and stature (Kruskal–Wallis: *H*_2_ = 22.751; *n* = 45; *p *< 0.001). A Bonferroni *post hoc* test indicates significant body mass differences between early *Homo* versus *Australopithecus* and *Paranthropus* (*p *< 0.001) but not between the latter two (*p *= 1.000). Mann–Whitney *post hoc* tests for the analyses of stature likewise show significant differences between early *Homo* versus *Australopithecus* and *Paranthropus* (*p *= 0.001). These findings are robust to different estimation methods (electronic supplementary material, text S3; table S3) and exclusion of potentially aberrant specimens (electronic supplementary material, text S2; table S1). Scatter plots of body mass and stature by time (figures 5 and 6) illustrate that the earliest fossils assigned to *Homo* in our sample (2.2–1.6 Ma) are generally heavier and taller, with little overlap to broadly contemporaneous *Paranthropus* or *Australopithecus*, even before the emergence of *Homo erectus s.l.* at *ca* 1.8 Ma.

**Figure 7.**
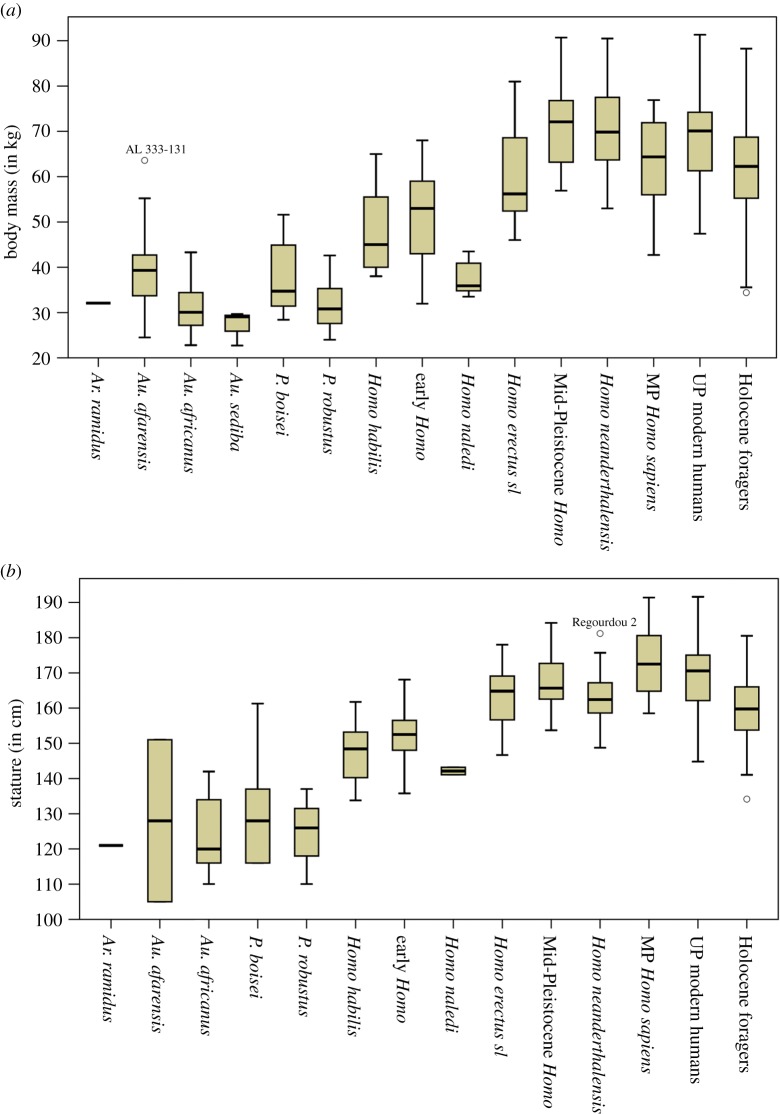
Body size estimates by narrow taxonomic groupings. (*a*) Body mass (in kg) and (*b*) stature (in cm).

Results for body size estimates by narrow taxonomic groupings (table 4 and figure 7) must be considered with care due to the unreliable species attribution for some taxa, particularly for those greater than 1.5 Ma [20,23]. Results for mean tendencies and variation in stature and body mass are, however, largely consistent with findings from the temporal analyses and broad taxonomic groupings (table 4 and figure 7; summary in electronic supplementary material, text S4).

**Table 4.** Taxonomic analyses of hominin body size estimates by fine groupings, ordered through time from younger to older.

taxonomic grouping	*n*	mean	median	mean CI L^a^	mean CI U^a^	s.d.	min	max	CV
body mass									
Holocene foragers^b^	438	61.86	62.28	—	—	10.71	34.39	88.23	17.3
UP modern humans	46	68.23	70.10	54.58	81.87	9.67	47.40	91.30	14.2
MP *Homo sapiens*	11	62.67	64.40	50.14	75.20	11.41	42.70	76.90	18.2
*Homo neanderthalensis*	30	70.52	69.85	56.42	84.63	9.14	53.00	90.50	13.0
Mid-Pleistocene *Homo*	31	70.47	72.10	56.34	84.58	9.19	56.90	90.70	13.0
*Homo erectus sl*	24	60.80	55.50	45.67	82.22	11.22	46.00	81.00	18.5
*Homo naledi*	8	37.53	35.90	30.02	45.03	3.75	33.50	43.50	10.0
early *Homo*	22	51.22	53.00	38.74	62.69	10.67	32.00	68.00	20.8
*Homo habilis*	7	48.43	45.00	37.39	61.71	10.42	38.00	65.00	21.5
*P. boisei*	11	38.07	34.70	23.83	61.14	8.18	28.40	51.60	21.5
*P. robustus*	9	31.70	30.80	18.48	51.31	6.14	24.00	42.60	19.4
*Au. sediba*	3	27.17	29.10	17.40	42.53	3.87	22.70	29.70	14.2
*Au. africanus*	24	31.07	30.05	20.73	50.02	5.04	22.80	43.30	16.2
*Au. afarensis*	18	39.94	39.30	26.08	61.64	10.36	24.50	63.60	25.9
*Ar. ramidus*	1	32.10	32.10	19.40	53.30	—	—	—	—
stature									
Holocene foragers^b^	449	159.91	159.74	—	—	8.28	134.16	180.55	5.2
UP modern humans	47	169.39	170.60	154.88	183.94	10.87	144.80	191.60	6.4
MP *Homo sapiens*	13	173.77	172.50	160.42	187.13	9.37	158.50	191.40	5.4
*Homo neanderthalensis*	38	162.66	162.40	152.05	173.24	7.25	148.70	181.20	4.5
Mid-Pleistocene *Homo*	32	166.90	165.70	159.07	174.68	6.95	153.70	184.20	4.2
*Homo erectus sl*	21	163.41	164.80	153.31	173.66	8.59	146.69	178.00	5.3
*Homo naledi*	2	142.15	142.15	134.40	151.75	1.48	141.10	143.20	1.0
early *Homo*	22	151.91	152.52	141.28	163.29	7.37	135.80	168.06	4.9
*Homo habilis*	7	147.26	148.37	138.91	156.11	10.03	133.82	161.73	6.8
*P. boisei*	6	131.05	128.00	112.50	137.50	16.97	116.00	161.30	12.9
*P. robustus*	3	124.33	126.00	111.90	136.77	13.57	110.00	137.00	10.9
*Au. africanus*	5	124.40	120.00	118.76	141.00	13.22	110.00	142.00	10.6
*Au. afarensis*	2	128.00	128.00	115.20	140.80	32.53	105.00	151.00	25.4
*Ar. ramidus*	1	121.00	121.00	117.00	123.00	—	—	—	—

^a^Lower and upper 95% confidence intervals of the mean (individual prediction intervals in electronic supplementary material, file S1).

^b^The Holocene forager sample is provided as comparative baseline and excluded from the statistical analyses.

### 3.3. Chronological variation within taxonomic categories

Temporal analyses within taxonomic categories test for diachronic change in body size among these groups. Linear regressions show a significant association of body mass and time in Neanderthals (*β* = −0.445; *p *= 0.014), early Mid-Pleistocene *Homo* (*β* = −0.826; *p *< 0.001) and australopithecines (*β* = 0.559; *p *< 0.001) among broad taxonomic groups, and for Upper Palaeolithic (UP) modern humans (*β* = 0.361; *p *= 0.014) and early *Homo* (*β* = 0.561; *p *= 0.007; 2.2–1.4 Ma) for the narrow taxonomy (electronic supplementary material, table S8). The majority of taxonomic groups exhibit no linear change. Regression slopes indicate a gradual increase of body mass throughout time in Neanderthals and early Mid-Pleistocene *Homo*, whereas australopithecines, early *Homo* and UP modern humans show a decline. For stature, only UP modern humans (*β* = 0.549; *p *< 0.001) and a combined lineage of Mid-Pleistocene *Homo* and Neanderthals (*β* = 0.294; *p *= 0.014) show a gradual decline over time (electronic supplementary material, table S8). All other groups do not exhibit significant associations of stature with time, suggesting within-group stasis.

Within-group increase in early Mid-Pleistocene *Homo* is driven by a rapid shift towards larger body mass after 0.5 Ma (electronic supplementary material, figure S7). This marked change is found in non-*Homo erectus* specimens (e.g. Arago, Boxgrove) and the SH hominins, which form the large-bodied ‘Mid-Pleistocene *Homo*’ group. These observations support a step increase in body mass within Mid-Pleistocene *Homo* after 0.5 Ma relative to late *Homo erectus*. UP modern humans exhibit a consistent decrease in stature and body mass through time, contrasting with Neanderthals that show an increase in body mass only (electronic supplementary material, figure S8). In sum, most taxonomic groups exhibit internal stasis with some taxa demonstrating gradual change over time.

## 4. Discussion

Consistent with previous studies [4,6–8,28,30], our long-term analyses of body size estimates by time indicate a significant positive association throughout the past 4.4 Myr. Hominins thus conform to Cope's rule like many other mammalian lineages [64–66]. There is, however, a high degree of variability for each analysed temporal group of hominins. Starting from a purely descriptive perspective, the pattern of increasing body size through time is interrupted by marked reduction in stature and body mass among australopithecines between 3.2 and 2.2 Ma and around 0.3 Ma coinciding with the late appearance of small-bodied *Homo naledi* specimens (figure 1). Within the age bracket of 3.2–2.2 Ma, species of *Australopithecus* (*africanus* and *sediba*) and *Paranthropus* (*boisei* and *robustus*) show smaller body mass and shorter stature compared with preceding *Australopithecus afarensis*, indicating more complex and potentially nonlinear chronological trajectories in earlier hominin evolution [23,24]. From around 2.2 Ma, the trend of increasing body size within the genus *Homo* continues [6–10]. While Middle and Late Pleistocene *Homo* are indistinguishable in their large body mass from one another, they exhibit significantly higher values compared with Early Pleistocene and Late Pliocene hominins. The exception to this general pattern is the retention of small body mass and stature in the late Middle Pleistocene individuals of *Homo naledi* and Late Pleistocene *Homo floresiensis*, which form clear outliers with significantly smaller body size compared to contemporaneous *Homo* specimens and taxa (see electronic supplementary material, figures S3 and S4). At the end of the Late Pleistocene, the analyses found a gradual within-group decrease of body size for UP modern humans.

Although an overall size increase throughout human evolution has long been accepted [4,6–8,28,30] the pattern and timing of changes in mass and stature have yet to be clarified within a large-scale perspective that takes both time and taxonomy into consideration. Our temporal analyses demonstrate that increases in body size throughout the past 4.4 Myr are not monotonic or gradual, but rather characterized by pulses of marked shifts or step changes. While chronological trends across lineages cannot be equated with evolutionary rates, the resulting pattern of relatively rapid increases within short timeframes is supported by within-group analyses that show stasis for many taxonomic units. As we detected some within-group trends—particularly in later *Homo*—long-term patterns of body size evolution in the hominin lineage can best be characterized by a mixture of processes in which dominant step changes are supplemented by less frequent gradual change (see also [40]).

The observed temporal patterns could be the result of various micro- and macro-evolutionary processes, acting independently or in combination [67]. Micro-evolutionary mechanisms include directional (anagenetic) change within lineages (i.e. gradual drift or selective shift between different means) resulting in a relatively continuous pattern of change through time. Our analyses suggest that such directional changes occur only within few taxonomic groups, whereas marked differences are often found *between* temporal and taxonomic units. Many palaeontological studies of vertebrates [65–70] have found that marked changes in body size distributions are often caused by macro-evolutionary processes such as speciation events (i.e. cladogenesis), species selection resulting from differential proliferation of taxa (e.g. larger-bodied taxa with higher fitness outcompeting smaller-bodied taxa; correlation of body size with species origination and/or extinction rates) or the extinction of small-bodied species within evolving lineages. Any of these macro-evolutionary processes could produce the punctuated net increases in size between the temporal groups found here. While discriminating between such interpretations requires testing with formal hominin phylogenies, the following combined discussion of temporal and taxonomic results can provide some initial insight into the evolutionary processes behind these patterns.

**Figure 8.**
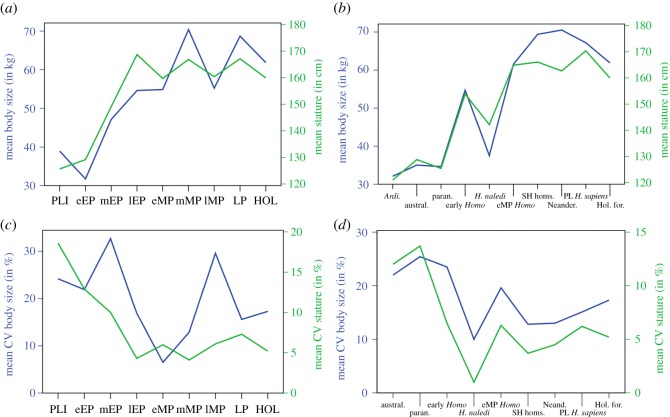
Summary of mean values and variation in hominin body size estimates. Double line graphs for: (*a*) mean body mass (in kg; blue) and stature (in cm; green) by fine temporal group; (*b*) mean body mass (in kg; blue) and stature (in cm; green) by broad taxonomic group; (*c*) coefficients of variation (in %) for body mass (blue) and stature (green) by fine temporal group; (*d*) coefficients of variation (in %) for body mass (blue) and stature (green) by broad taxonomic group. PLI, Late Pliocene; eEP, early Early Pleistocene; mEP, middle Early Pleistocene; lEP, late Early Pleistocene; eMP, early Middle Pleistocene; mMP, middle Middle Pleistocene; lMP, late Middle Pleistocene; LP, Late Pleistocene; HOL, Holocene.

Regarding the larger and statistically significant punctuated shifts in body size parameters through time, we found a complex pattern with separate temporal trajectories for body mass and stature in the Pleistocene (figure 8): marked increases in both stature and mass (*ca* 2.2–1.9 Ma) are followed by a sole increase in stature (1.6–1.4 Ma), and only later by increases in mass (*ca* 0.5–0.4 Ma). The first step change coincides with the earliest fossils of *Homo* in our sample—as well as a decreasing number and last appearance datum of several australopithecines—and might thus result from a mixture of cladogenesis and the extinction of smaller-bodied forms. This period of change is followed by a major shift in stature already in the late Early Pleistocene, long before respective increases in body mass. The second major increase in body mass is found only at *ca* 0.5–0.4 Ma, coinciding with the SH hominin sample and contemporaneous non-*Homo erectus* specimens (electronic supplementary material, figure S7; see also [13]), and associated with migrations to higher latitudes (see below).

New postcranial fossils from currently underrepresented time periods—such as 3.1–2.2 Ma and the late Early Pleistocene—will be required to test the temporal patterns of body size evolution found here. Additional data on ancestor–descendant relationships—which are at the moment without consensus [20,23,26,27,29,37,71–74]—are also needed to formally test evolutionary rates of body size changes within and between lineages, control for the effects of phylogenetic inertia and assess the impact of potential lineage extinctions. These issues are most evident for the small-bodied *Homo naledi* specimens dating to *ca* 0.3 Ma which lie closest in body size to australopithecines (for body mass) and early *Homo* (for stature), but are significantly smaller than contemporaneous *Homo* and are also more similar to the former taxa in their overall morphology [75–77]. Only further phylogenetic analyses will be able to answer whether the small body size of this species is due to a retention of ancestral small body size, hinting at a long and hitherto unrecognized persistence of archaic traits in postcranial build, or rather a convergent trait that evolved anew in this lineage due to localized selection pressures. The existence of even smaller-bodied individuals of *Homo floresiensis* in the Late Pleistocene raises similar questions and shows that this is not an isolated case.

This study also found marked differences in absolute and relative variability in body size throughout time that correspond with broad taxonomic units. The results should be treated carefully as estimates of variation are more unreliable than central tendencies [61], and some of the temporal groups include variable numbers of independent populations at different time points. The incomplete nature of the fossil record also implies that some extinct populations have not been sampled and additional biases (e.g. preservation in different habitats) are difficult to assess in relation to body size variability [62]. Our method may have also increased the amount of variation observed compared with studies which use a consistent estimation method on a single skeletal element only. This being said, testing the effect of the use of different methods and number of studies involved per analytical unit did not change the observed relative patterns of variation to a large extent (see electronic supplementary material, text S3; tables S2 and S3).

Regardless of estimation methods, consistently high values of relative size variability characterize Pliocene and Early Pleistocene hominins, encompassing *Australopithecus*, *Paranthropus* and early *Homo* (figure 8*c,d*). Previous studies have associated the high variability of older species—including taxa of early *Homo*—with higher sexual dimorphism [71,78–80], particularly in *Australopithecus afarensis* [23,24,78,79]. Variability in body size is markedly reduced after the (middle) Early Pleistocene and continues at comparably low levels in the Middle and Late Pleistocene (figure 8*c*), with the late Middle Pleistocene breaking this pattern due to the occurrence of both very large (SH hominins) and small-bodied taxa (*Homo naledi*) in this age bracket. A generally reduced size variability after the (middle) Early Pleistocene corresponds with observations that—with the exception of *Homo floresiensis* (LB1) and *Homo naledi*—no fossil Pleistocene hominins of small body size (less than 40 kg; less than 140 cm) occur after 1.4 Ma (figure 1), suggesting active selection against this ancestral trait and the extinction of most small-bodied lineages. Accordingly, our taxonomic analyses show that among *Homo,* younger species tend to be less variable in body size than older taxa (figure 8*d*), potentially associated with decreasing sexual dimorphism. These findings demonstrate abundant change in variability, but no consistent linear pattern of increasing or decreasing variation throughout time. The entire variability spectrum of present humans is only attained with the Holocene foragers.

We conclude that there have also been two major shifts in body size variability, which do not easily correlate with transitions in mean size. The first shift takes place between the Early and Middle Pleistocene—within the earlier part of the genus *Homo*—during which a selection against smaller body mass and stature occurred in most taxa (i.e. proliferation of larger-bodied *Homo erectus s.l*. and Mid-Pleistocene *Homo*), with the extinction and replacement of smaller-bodied taxa (e.g. early *Homo*; *Homo habilis*). This process narrowed the range of body sizes by shifting the overall spectrum towards larger bodies, with *Homo naledi* and *Homo floresiensis* as notable deviations from the general pattern. The second transition occurred in the Holocene, with higher absolute diversity and reintroduction of small body shapes corresponding with global colonization and concomitant adaptations to virtually all of the world's environments, including secondary adaptations to islands and rainforests [81,82].

The high variability in body sizes during the middle Early Pleistocene (2.0–1.4 Ma) supports our previous findings of large-scale temporal (and spatial) heterogeneity within early *Homo* [20]. Our findings correspond with high variability in morphology and size of cranio-dental remains for this time frame in general [83], and early *Homo* in particular [29,30,71,84,85] also for postcranial material [18,19]. Data on taxonomic groupings (tables 3 and 4) demonstrate that body size variability in the chronological analyses is not exclusively a result of taxic diversity within particular timeframes, but also due to higher intra-taxic variation in earlier hominin groupings (greater than 1.0 Ma). These observations highlight the importance of studying intra-taxon variability and point to a potentially elevated role of phenotypic plasticity in the evolution of early *Homo*, as well as *Homo erectus s.l.* [30,34,51,52].

Combined temporal and taxonomic analyses of changes in body size have the potential to shed new light on other debated issues in human evolution. Previous studies have either placed a marked body size increase close to the origin of *Homo* (in early *Homo* or ‘non-*erectus* early *Homo*’) [21,22,28,30,39,71,86] or only later with the evolution of *Homo ergaster* and sometime after 2.0 Ma [23,24,26]. The various tests for both body mass and stature of this study are consistent with previous results in that the origins of *Homo* are characterized by a significant increase in body size compared with *Australopithecus* and *Paranthropus*. Early *Homo* specimens between 2.2 and 1.6 Ma—excluding those assigned to *Homo erectus/ergaster*—show a marked and significant increase in body mass and stature compared with the broadly contemporaneous postcranial fossils of *Paranthropus boisei* (*ca* 1.9–1.3 Ma)*, Paranthropus robustus* (*ca* 1.9 Ma) and *Au. sediba* (1.977 Ma) as well as to the slightly earlier *Australopithecus africanus* (*ca* 2.8–2.2 Ma). The difference between early *Homo* specimens and *Au. afarensis* is muted, although there is a temporal gap of at least 900 000 years between the fossils of this study (figures 5 and 6). These results remain consistent when excluding fossils that have yielded large differences in body size estimates (greater than 30%) between key studies [8,20,23] for this time frame [24] (electronic supplementary material, text S2 and table S1), and when controlling for potential bias introduced by using different estimation methods (electronic supplementary material, text S3 and tables S2 and S3).

Although the oldest representatives of *Homo* in our analyses are significantly larger in body size compared with contemporaneous and immediately preceding australopithecines, the lack of postcranial fossils and body sizes estimates for earliest *Homo* between 2.8 and 2.3 Ma—known exclusively from cranio-dental fossils [27,30,87]—could mask a more gradual transition in body size and shape [31], and the full ancestral diversity in body size of australopithecines might be obscured by a biased fossil record. At the same time, fossils of early *Homo* between 2.2 and 1.5 Ma show the highest relative variation of body sizes among their genus (see also [20,30]). They also still feature several small-bodied individuals (less than 140 cm; less than 40 kg; figure 1) which are absent after 1.4 Ma in *Homo* taxa with the exception of *Homo naledi* and *Homo floresiensis*. Regarding diversification in body size and shape, early members of *Homo* are closer to *Australopithecus* and *Paranthropus* than to later *Homo* (see also [23,24]).

The last large-scale study dedicated to the evolution of body size within *Homo* [10] identified a major increase in average body size with the emergence of *Homo erectus s.l.* followed by a long period of relative stasis (see also [23,30,71]). Our taxonomic analyses of an enlarged sample identified similar patterns, but with two major shifts in different body size parameters. A first marked increase in stature—and a minor one in body mass—took place between early *Homo* (including *Homo habilis*) and *Homo erectus s.l*. after 1.7 Ma (see also [15,23–25,30]), but is difficult to pinpoint in time due to the large amount of spatial and temporal variability (figures 5 and 6; see also [20]), which is also reflected in relatively high CVs in body mass for *Homo erectus s.l.* compared with later *Homo* (table 4)*.* The second major increase, exclusively in body mass, occurs among later Mid-Pleistocene *Homo*, and more particularly in the SH palaeo-population and liked-aged non-*Homo erectus* specimens (see also [13]). This decoupling in body size parameters corresponds with the temporal results, suggesting an earlier increase of stature in *Homo erectus s.l.* around 1.6–1.4 Ma, followed much later by significantly larger body masses (figure 8*a*,*b*).

Calculation of ponderal and body mass indices as proxies for body form [24] (figure 6; electronic supplementary material, figure S9 and tables S9–S12) better illustrates the interplay between the two body size parameters and helps to assess the evolutionary processes behind these patterns. The ponderal index shows a slight decrease throughout time, but with taxonomic differences among later *Homo*. Early Mid-Pleistocene *Homo* (e.g. *Homo erectus*) and Pleistocene *Homo sapiens* show consistently low values, with the minimum reached by predominantly African MP *Homo sapiens*. By contrast, Eurasian Mid-Pleistocene *Homo* and particularly Neanderthals exhibit high ponderal and body mass indices (figure 6; electronic supplementary material, figure S9 and tables S9–S12), highlighting continuity and long-term phenotypic evolution within this lineage. Later *Homo* species thus retained the tall stature from *Homo erectus s.l.*, but increased their body mass markedly. These different trajectories in body size parameters indicate a directional selection towards greater body mass in Middle Pleistocene *Homo* that fits eco-geographical predictions associated with migration to higher latitudes and climatic adaptations according to Bergmann's rule, involving thermoregulation as a selective pressure on hominin phenotype [86,88,89]: the majority of the younger sample (less than 0.5 Ma) in our study derives from Europe (70%), whereas older hominin fossils are mostly of African or southern Asian origin (97%). Directional selective pressure towards larger body mass in later *Homo* is also supported by temporal analyses within Neanderthals (electronic supplementary material, figure S8). Late Pliocene and Early Pleistocene hominins are characterized by more variable body forms (i.e. higher CVs; see electronic supplementary material, tables S9–S12).

Our study adds new perspectives to hominin body size evolution with its focus on long-term and inter-taxonomic patterns and variability through time in both body mass and stature. The results indicate complex temporal patterns of body size and size variability across—and sometimes within—hominin taxa, which could be due to various micro- and macro-evolutionary processes [67] that need to be resolved by further comparative phylogenetic analyses. Long phases of stasis indicate only minor anagenetic increases within many taxonomic groups analysed here (e.g. *Homo erectus*; *Australopithecus africanus*). The marked and seemingly rapid shifts in size through time, particularly in the earlier Early Pleistocene, could be the result of cladogenesis (i.e. the emergence of *Homo*), the differential proliferation of large-bodied taxa or the extinction of smaller-bodied forms at certain points in time (i.e. small-bodied australopithecines) as observed for many other vertebrates and mammals (e.g. [65,69,70]). Stature and mass evolution follow different trajectories in later *Homo* (figure 8), probably due to directional selection on larger body masses associated with increased migration of Mid-Pleistocene hominins to higher latitudes that are frequent in our sample. The findings suggest selection against small body size operating from *ca* 1.4 Ma—associated with the extinction of most small-bodied taxa—but especially evident in the Middle and Late Pleistocene. Maintenance of larger bodies and reduced variability on the population level in late Early and Middle Pleistocene *Homo* could be the long-term result of inter-species competition accompanied by a shift in ecological niches, changes in dietary behaviour (higher quality diets; carnivory), locomotor patterns, body proportions and loading patterns, or other energetic and life-history factors [28,30,90–92], adaptation to increased environmental variability [30,50,93] or a decrease in sexual dimorphism due to behavioural changes [78,79]. While the reported trends in later *Homo* apply to most recognized taxa, the appearance of small-bodied individuals in the late Middle Pleistocene (*Homo naledi*) and Late Pleistocene (*Homo floresiensis*) suggests additional layers of complexity to the evolution of body size in the genus *Homo*. This diversity might derive from ancestral retentions, the operation of more localized selection pressures with convergent evolution of small physique or neutral drift that operated particularly in small and isolated populations [74].

Beside issues of causality, other open questions and challenges remain. The scarcity of postcranial fossils for crucial periods—for *Homo* between 2.8 and 2.0 Ma as well as the late Early Pleistocene and late Middle Pleistocene—remains a significant challenge, as are taxonomic attributions for many fragmentary specimens greater than 1.0 Ma. The recent finding of small-bodied *Homo naledi* in the late Middle Pleistocene serves as a case in point [48,49,75–77] and hints at even more complex and unexpected patterns as the fossil record increases. While one of our main goals was to increase sample size as much as possible in this study—with the trade-off that this approach necessarily includes estimates based on different studies, methods and elements that are not all of the same accuracy—future discoveries and larger-scale analyses will be required to more broadly test our results.

We are also in much need for more reliable and comparable methods to estimate body size [14,16,20,23,25,42–46] applicable to various genera of hominins [45,61], particularly when they exhibit different body proportions and lack the secular growth effects of modern-day human reference samples. Using populations with small-bodied individuals from hunter–gatherer populations [20], working with more advanced multivariate models among large samples [23] or applying non-allometric approaches such as a morphometric methods [45,94] or a convex hull-based volumetric technique [46] can be considered as first steps in this direction, and we have included the novel data resulting from these methodical advancements wherever possible. Confidence intervals for body size estimates should be specified, both for individual estimates (electronic supplementary material, file S1) and mean values for temporal and taxonomic groups (tables 1–4; also [23]), to show the prediction error involved in all methods of body size estimation [61]. Increasing sample size for analytical categories is crucial to distinguish between noise and pattern in hominin body size variation [20], and this was one of the primary motivators underlying this study. Nevertheless, methodical advancements will be necessary (e.g. for fragmentary remains or non-lower limb elements) to increase the quality and commensurability of estimates.

Our results have important ramifications for studies concerned with human energetics, dispersal and encephalization, but also more generally for how we interpret the evolution and biology of our genus. In particular, this study underscores the large variability in body size in the hominin lineage and the complex pattern of its evolution throughout time and among taxa. Rather than focusing exclusively on species means and unidirectional models, perspectives that emphasize variation and nonlinear patterns within multidirectional trajectories might thus be fruitful strategies for interpreting the evolution of body size in our lineage. Such an approach could also work within the framework of phenotypic plasticity that might explain some of the observed variation in relation to specific natural environments, adaptive strategies and cultural capacities, particularly around the origin of our genus [30,34,51,52].

## Supplementary Material

Supplementary Information

## Supplementary Material

Supplementary file 1

## Supplementary Material

Supplementary file 2

## Supplementary Material

Supplementary file 3

## Data accessibility

All of our raw data and materials are available as electronic supplementary material (electronic supplementary material, file S1) or are part of this paper.
