# Supplementary material for: Long-term patterns of body mass and stature evolution within the hominin lineage
Source: R Soc Open Sci. 2017 Nov 8;4(11):171339. doi: 10.1098/rsos.171339 (PMC5717693; doi:10.1098/rsos.171339)
Supplement: Supplementary Information [file rsos171339supp1.docx]

**SUPPLEMENTARY INFORMATION**

**Article: “Long-term patterns of body mass and stature evolution within the hominin lineage”**

Manuel Will, Adrián Pablos, Jay T. Stock

*Corresponding author: Manuel Will (*[*Manuel.Will@uni-tuebingen.de*](mailto:Manuel.Will@uni-tuebingen.de)*;* [*mw540@cam.ac.uk*](mailto:mw540@cam.ac.uk)*)*

**Supplementary Files**

**Supplementary File 1**

The database of hominin body size estimates in Excel format is attached separately.

**Supplementary File 2**

The database of Atapuerca hominins ordered by different selection criteria in Excel format is attached separately.

**Supplementary File 3**

The dataset for the test of major chronological and taxonomic body size changes regarding both mean values and coefficients of variation in Excel format is attached separately.

**Supplementary Text**

**Supplementary Text 1 – Selection criteria for the Atapuerca hominins**

The Sima de los Huesos (SH) human collection from the Sierra de Atapuerca (Burgos, Spain) likely belonged to the ancestral Neanderthal clade (Arsuaga et al., 2014; 2015). These human fossils were recovered from lithostratigraphic unit 6 (LU6) dated to ~430 ka (Arsuaga et al., 2014; Aranburu et al., 2017). In the SH collection, the skeletons of at least 28 individuals were identified based on dental evidence (Bermúdez de Castro et al., 2004b). Regarding the aims of the current study, the biggest challenge with the SH collection is the mixed nature of the fossil material, complicating the attribution of different postcranial elements to the same individual (Pablos et al., 2013; 2014; Arsuaga et al., 2015). In order to avoid repetitive and misleading estimates within the SH collection, we established several criteria for rendering the body mass and stature estimates to be more reliable and representative of the paleo-population at the site:

**Criterion 1.** Body size estimates obtained from lower limb bones, especially those directly involved in stature and body mass (femur, coxal, tibia and fibula). Estimates of body size from the lower limbs are generally more reliable than those obtained from the upper limb (see for example Carretero et al., 2012; Pablos et al., 2013). The sample size of this group is 20 elements, amounting to n=11 estimates of body mass and n=11 estimates of stature.

**Criterion 2.** Body size estimates obtained from the secure foot associations among the SH collection (average of estimates from different tarsal bones). This increases the overall sample size to 26 elements (n=15 body mass; n=18 stature). The sample size can be expanded by including the "SH foot association 2" composed of 2 tali and 2 calcanei. The tali are morphologically adult (>14 years old), the right calcaneus is finishing the fusion of the tubercle, and the left calcaneus is fully adult. Pablos et al. (2013; 2014) established a biological age of around 17 years old for this female individual, bringing the overall sample size to 27 elements (n=16 body mass; n=18 stature).

**Criterion 3.** Body size estimates obtained from isolated foot elements with known sex. In this case, overall sample size increases to 33 elements (n=21 body mass; n=24 stature).

**Criterion 4.** Body size estimates obtained from isolated foot remains that are either incomplete or eroded and with unknown sex. Measurements for body size estimates are not affected. Overall sample size = 42 elements (n=26 body mass; n=30 stature).

**Criterion 5.** Body size estimates obtained from long bones of the upper limb (humerus, radius, ulna). Sample size = 56 elements (n=26 body mass; n=44 stature).

The attached Excel file (Supplementary File 2) shows the five groups from SH according to the different criteria. For this study, we chose as a good equilibrium to include only those elements that belong to the first four criteria, excluding estimates from the upper limb (Criterion 5). This leads to a total number of estimates from the SH collection of n=26 for body mass and n=30 for stature used in this study.

**Supplementary Text 2. Test for comparability and robusticity of results**

In order to test the comparability and robusticity of our analyses, individual fossils for which body size estimates diverged strongly between different key studies (>30% body mass; 20% stature) were removed (see Supplementary Table 1), and the remaining sample analyzed separately. Results of this approach were checked for consistency against results deriving from the entire database, to ensure that the diverging estimates do not bias the overall analysis. The overwhelmingly majority of the analyses on these reduced sample yields comparable results to those from the entire database (e.g. finding the same statistically significant differences), with only minor changes in measures of central tendency (between 0.1-2.7 kg changes in mean and median for any taxonomic or temporal group with reduced sample size) and variation (<2% changes in CVs). These changes do not affect any of the main findings. We therefore report only the significant differences in the following.

*Body mass*

The comparative analysis for body mass was performed by the removal of n=12 estimates. In comparison to the result from the entire database, this analysis found major differences for the fine chronological groups only. Here, the largest disparity is expected to come from the middle Early Pleistocene as its sample size is reduced the most (n=10). A Kruskal Wallis test shows that in contrast to the results for the entire sample, the shift between early Early Pleistocene (31.6 kg) and middle Early Pleistocene (45.8 kg) is now not significant anymore when adjusted for multiple comparisons (p=0.348). The mean body size of this latter group, however, is only 1.3 kg less than before. The results are thus likely influenced by lower sample sizes as the mere body mass values still show a large gap of ca. 14.2 kg between the groups (compared to 15.4 kg before).

*Stature*

The comparative analysis for stature was performed by the removal of n=1 estimate (OH 62). In comparison to the result from the entire database, the analyses without this estimate did not find any major or significant differences to the main findings.

**Supplementary Text 3. Test of major chronological and taxonomic body size changes**

**a) Mid-Pleistocene *Homo:* body mass increase after 0.5 Mya**

Data & methods

The study identified a marked step increase in body mass after 0.5 Mya that coincides with the SH hominins and other Eurasian Mid-Pleistocene *Homo* specimens (e.g. Arago; Boxgrove). From a methodological perspective, most of the estimates before this observed change (1.5-0.6 Mya) come from Will & Stock (2015) and Grabowski *et al.* (2015), while the majority of estimates between 0.5-0.3 Mya derive from McHenry (1992) and Arsuaga et al. (2015) which used diverging estimation methods (Supplementary File 1: “Details of Methods”). To evaluate whether the step increase around 0.5 Mya is merely the result of different methodologies, we estimated body mass for specimens between 0.5-0.3 Mya (the “middle Middle Pleistocene” including the SH hominins and “Mid-Pleistocene *Homo*” in the broad and narrow taxonomy respectively) with the methods and regression formulae described in Will & Stock (2015) to compare estimates deriving from the same methodology. The detailed approach and results can be found in Supplementary File 3 (“Test Mid-Plei. *Homo*”). Stature was also tested to assess whether measures of variability (CVs) are affected by using different methods of body size estimation. Here we report on overall descriptives and statistical tests (see Supplementary Table 2).

Results

The descriptives show that using the predictions by Will & Stock yields overall comparable values for the relevant taxonomic and chronological groups that are on average ~4 kg lighter and 5 cm shorter (Supplementary Table 2a). The CVs are likewise similar, with differences for both body mass and stature ranging only between -1.0 to +2.2. This suggests that the low CVs for SH hominins and the middle Middle Pleistocene (particularly in comparison to earlier taxonomic and chronological groups) are not the result of different methodologies but actual trends of decreasing body size variability. A comparison of the new average values (Supplementary Table 2b-1) with means in the study of the immediately preceding chronological and taxonomic groups show that there is still a marked increase between the early Middle Pleistocene vs. middle Middle Pleistocene (+11.4 kg). The notion of this step increase is further supported by statistical tests using the newly estimated body mass for specimens <0.5 Mya. Here, a Kruskal-Wallis test between the fine chronological groups find that the difference between early Middle Pleistocene vs. middle Middle Pleistocene is still significant (p=0.033). While differences in the taxonomic results are muted, there remains an increase of over 5 kg on average between *Homo erectus s.l.* and Mid-Pleistocene *Homo* (Supplementary Table 2b-3).

**b) Body size increase of early *Homo* compared to australopithecines**

Data & methods

The study identified a significant increase in body mass and stature in early *Homo* compared to preceding *Australopithecus* and *Paranthropus*. From a methodological perspective, however, most of the estimates for *Australopithecus* and *Paranthropus* come from Grabowski *et al.* (2015) and McHenry (1991; 1992), while the early *Homo* predictions derive almost exclusively from Will & Stock (2015), who used diverging methods (Supplementary File 1 “Details of Methods”). To evaluate whether the size increase between *Australopithecus/ Paranthropus* vs. *Homo* is dependent on different methodologies or not, we estimated body mass and stature for all *Australopithecus* and *Paranthropus* specimens with the methodology and regression formulae described in Will & Stock (2015). The detailed approach and results can be found in Supplementary File 3 (“Test Austral.”). In addition to comparing mean values, we also tested whether measures of variability (CVs) are affected by using different methods of body size estimation. Here we report on overall descriptives and statistical tests (see also Supplementary Table 3). We emphasize that we used this approach only for test reasons and advise that our regression equations (based on modern human hunter-gatherers) should only be applied with great care to australopithecines of likely smaller body size and different body proportions as they will tend to overestimate the true sizes (see discussion in Grabowski et al. 2015).

Results

The descriptives show that using the predictions by Will & Stock yields overall comparable values for *Australopithecus* and *Paranthropus* (Supplementary Table 3a), but consistently higher means (body mass: +4-5 kg; stature: +8-11 cm) as expected from methodological considerations (see above). CVs for *Australopithecus* and *Paranthropus* differ by only +2.0% and -0.3% respectively for body mass, but between -4.8% and -6.4% for stature. Particularly for body mass in individual species, many of the CVs reached by the Will & Stock predictions show even higher values (17.0-25.9%) supporting the high variability found in the study, based on the estimates by Grabowski *et al.* (2015) and McHenry (1992). For stature, the CVs resulting from Will & Stock are generally lower compared to McHenry’s (1991) estimates, ranging between 5.4-16.8%, and particularly low for *Paranthropus*. For *Australopithecus*, however, CVs are still higher than for all other *Homo* groups.

Comparing the new average values of *Australopithecus* and *Paranthropus* (Supplementary Table 3b-1) with means of early *Homo* (excluding *Homo erectus s.l*.) using the same regression formulae (in the study) shows that while size differences are smaller compared to the values in the study, there is still a marked increase between the early *Homo* vs. *Australopithecus* (body mass: +12.4 kg; stature: +14.7 cm) and vs. *Paranthropus* (body mass: +11.7 kg; stature +13.8 cm).

As in the study, statistical comparisons identify significant differences between the three groups regarding both body mass (ANOVA: F(2,94)=12.787; p<0.001) and stature (Kruskal-Wallis: H(2)=16.684; n=45; p<0.001) even when the new and larger estimates for *Australopithecus* and *Paranthropus* are applied. A Bonferroni post-hoc test indicates significant body mass differences between early *Homo* vs. *Australopithecus* and *Paranthropus* (p<0.001) but not between the latter two (p=0.994). Post-hoc tests for the analyses of stature likewise show significant differences between early *Homo* vs. *Australopithecus* (p=0.004) and vs. *Paranthropus* (p=0.001). A significant increase in body size within *Homo* compared to preceding australopithecines is found when both groups are estimated via the Will & Stock regression equations, and thus not contingent on the particular method used for size estimates in this study (e.g. bias introduced by using comparatively low values for australopithecines estimated by Grabowski et al. (2015)).

**Supplementary Text 4. Summary of results narrow taxonomic groupings**

Concerning results for body mass by narrow taxonomic groupings, summary statistics, box plots and scatter plots suggest four groups of body size (Table 4; Figure 7): 1) *Ar. ramidus* plus species of *Australopithecus*, *Paranthropus* and *Homo naledi* (mean: 27.2-39.9 kg; median: 29.1-39.3 kg); 2) Early *Homo* and *Homo habilis* (mean: 48.4-51.2 kg; median: 45.0-53.0 kg); 3) *Homo erectus* s.l. (mean: 60.8 kg; median: 55.5 kg); 4) Mid-Pleistocene *Homo, Homo neanderthalensis,* MP *Homo sapiens*, UP modern humans (mean: 62.7-70.5 kg; median: 64.4-72.1 kg). The recent hunter gatherer sample (mean=61.9 kg; CV=17.3%; n=438) falls closest to MP *Homo sapiens* both in terms of means and relative variation. Corresponding results are reached for stature (Table 4; Figure 7), with the main difference that *Homo erectus s.l*. (mean=163.4 cm) groups firmly with Mid-Pleistocene *Homo* (mean=166.9 cm), *Homo neanderthalensis* (mean=162.7), MP *Homo sapiens* (mean=173.8 cm) and UP modern humans (mean=169.4 cm). Our recent hunter gatherer sample also fits best in this group, although at its lower end (mean=159.9 cm). Similar to body mass, stature estimates for *Homo naledi* falls at the lowest end of the genus *Homo*, but they are still >10 cm above taxa assigned to Australopithecus or Paranthropus.

Figures 5 & 6 (in the manuscript) give an overview on the variability of body mass and stature estimates by time and narrow taxonomic group attribution. Intra-group variation for body mass (Table 4) is particularly high for *A. afarensis* (CV=25.9%), *Paranthropus boisei* (CV=21.5%) and *Paranthropus robustus* (CV=19.4%), followed by earlier forms of *Homo* (early *Homo*, *Homo habilis*, *Homo erectus s.l.*; range of CVs=18.7-21.5%). More recent hominin groups including Mid-Pleistocene *Homo* (CV=13.0%), *Homo naledi* (CV=10.0%) *Homo neanderthalensis* (CV=13.0%), and UP modern humans (CV=14.2%) exhibit lower values that lie below the highly diverse Holocene foragers (CV=17.3%). MP *Homo sapiens*, however, do not fit into this temporal pattern with a CV (18.2%) that lies closest to Early Mid-Pleistocene *Homo* and above the Holocene group. For stature, all narrow taxonomic groupings of *Homo* show low relative variation with *Homo habilis* possessing the highest (CV=6.8%) and Mid-Pleistocene *Homo* (CV=4.2%) and *Homo naledi* (CV=1.0%, but n=2) the lowest values. All species of *Australopithecus* (CV=10.6-25.4%) and *Paranthropus* (CV=10.9-13.0%) exhibit larger intra-group variation.

**Supplementary Figures**

**Supplementary Figure 1. Line plot of mean body mass estimates by time in Mya.**

Body mass estimates for all fossil hominins regardless of species were averaged by increments of 100k years (e.g., 0.400-0.499 ka = 0.4 ka) and the mean plotted. Only 100k-intervals with a sample size of n>2 are included, with the oldest such sample at 3.4 Mya. The grey rectangles indicate missing data, with the lines within grey boxes representing interpolations between adjacent 100k-intervals. There are at least two rapid increases between ca. 2.2-1.9 Mya and particularly marked after 0.6 Mya. The sharp drop in body mass estimates at ~0.3 Mya is caused entirely by the small-bodied *Homo naledi* specimens dated to ~286 ka. Note, however, that different taxonomic units make up some of the 100ky-intervals, thereby influencing the estimates particularly between 2.0-1.5 Mya (e.g. larger-bodied *Homo* and smaller-bodied *Paranthropus*).

***
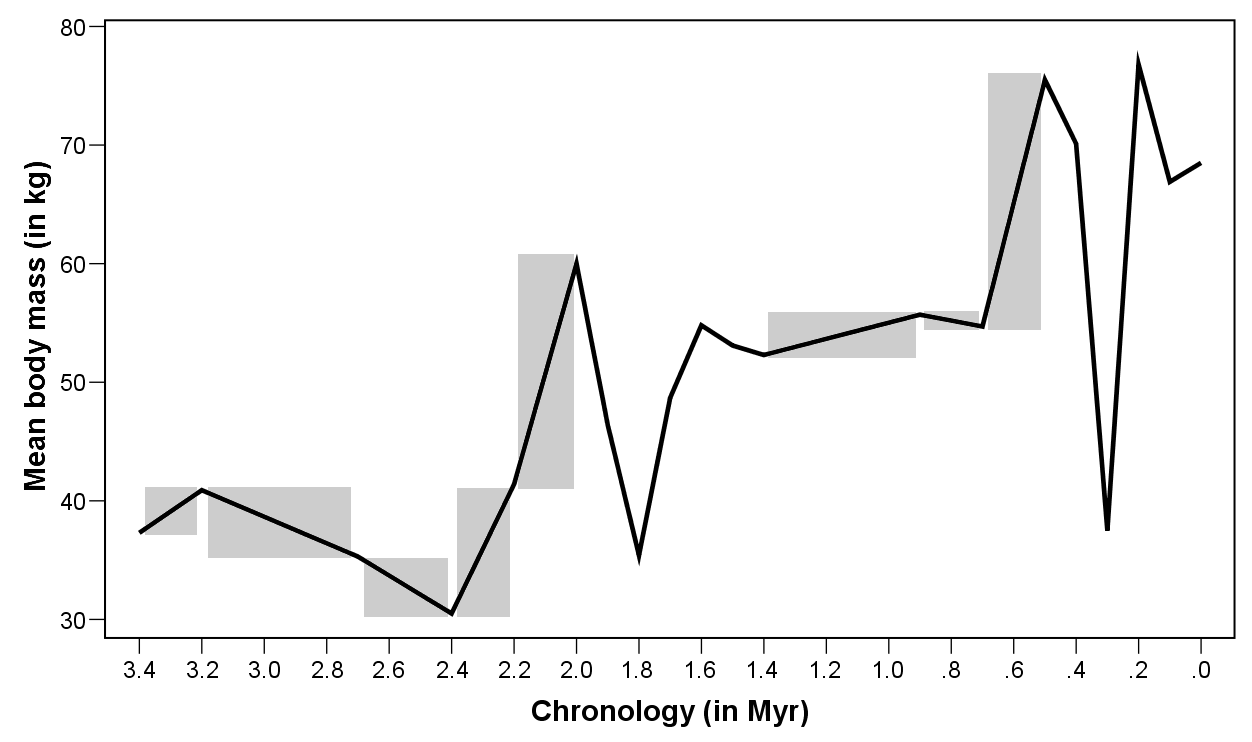
***

**Supplementary Figure 2. Line plot of mean stature estimates by time in Mya.**

Stature estimates for all fossil hominins regardless of species were averaged by increments of 100k years (e.g. 0.400-0.499 ka) and the mean plotted. Only 100ky-intervals with a sample size of n>2 are included, with the oldest such sample at 3.2 Mya. The grey rectangles indicate missing data, with the lines within grey boxes representing interpolations between adjacent 100k-intervals. There is at least one rapid and marked shift between 2.2-1.9 Mya. The sharp drop in stature estimates after 0.3 Mya is caused by the small-bodied *Homo naledi* specimens dated to ~286 ka. Note, however, that different taxonomic units make up some of the 100ky-intervals, thereby influencing the estimates particularly between 2.0-1.5 Mya (e.g. larger-bodied *Homo* and smaller-bodied *Paranthropus*).


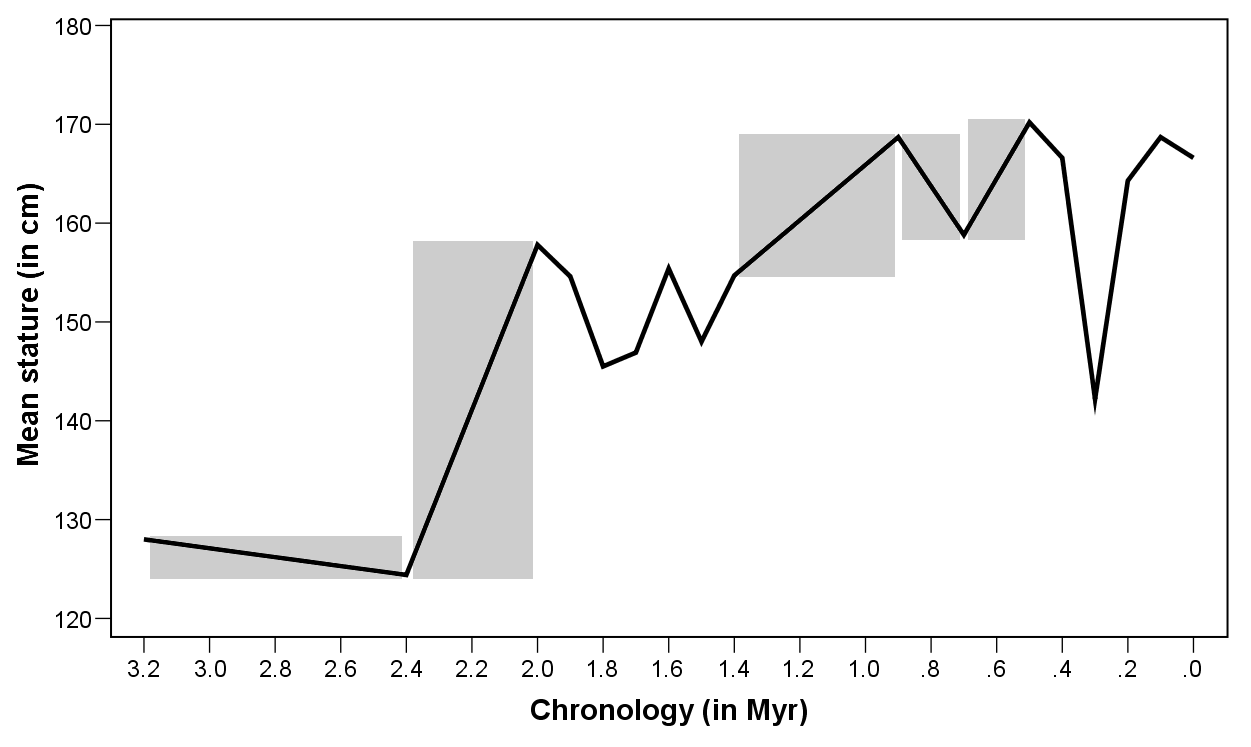


**Supplementary Figure 3.** Scatter plots of body mass estimates (in kg) by coarse temporal grouping with attribution to narrow taxonomic unit for each individual prediction. Note the outlier position of *Homo naledi* estimates within the Middle Pleistocene and *Homo floresiensis* (LB1) for the Late Pleistocene**.**

**
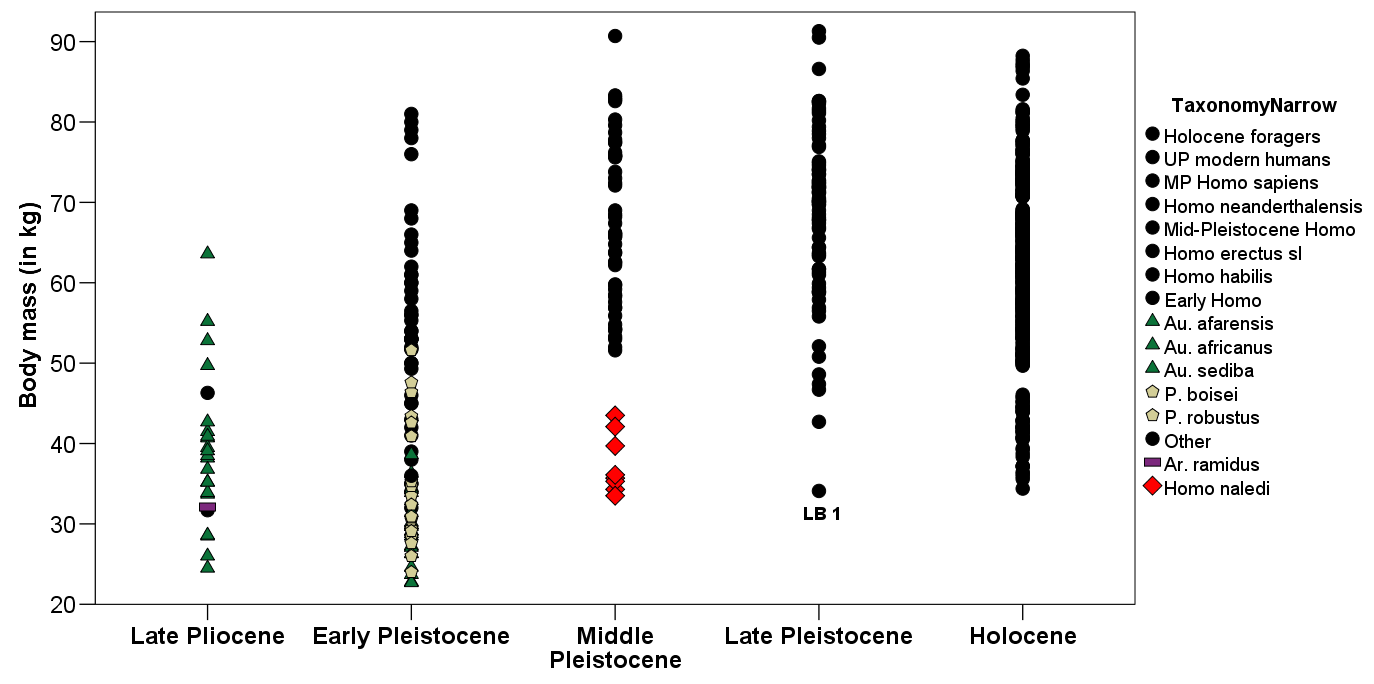
**

**Supplementary Figure 4.** Scatter plots of body mass estimates (in kg) by fine temporal grouping with attribution to narrow taxonomic unit for each individual prediction. Note the outlier position of *Homo naledi* estimates within the late Middle Pleistocene and *Homo floresiensis* (LB1) for the Late Pleistocene**.**

**
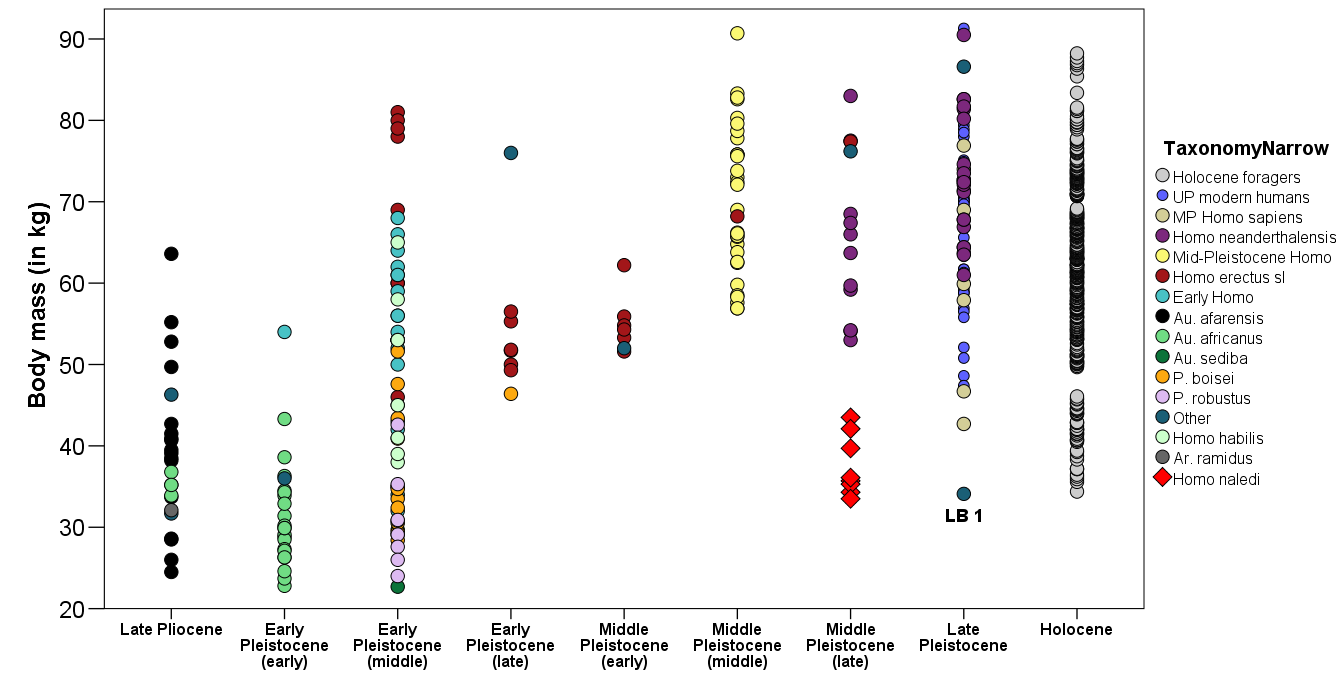
**

**Supplementary Figure 5. Body mass estimates by time with temporal group boundaries**

Scatter plot of body mass estimates for all fossil hominins by time with indication of membership of narrow taxonomic group. *Top*: Coarse temporal groups; *bottom:* Fine temporal groups. Abbreviations: EP=Early Pleistocene; eMP=early Middle Pleistocene; mMP=middle Middle Pleistocene; lMP=late Middle Pleistocene; LP=Late Pleistocene; H=Holocene.


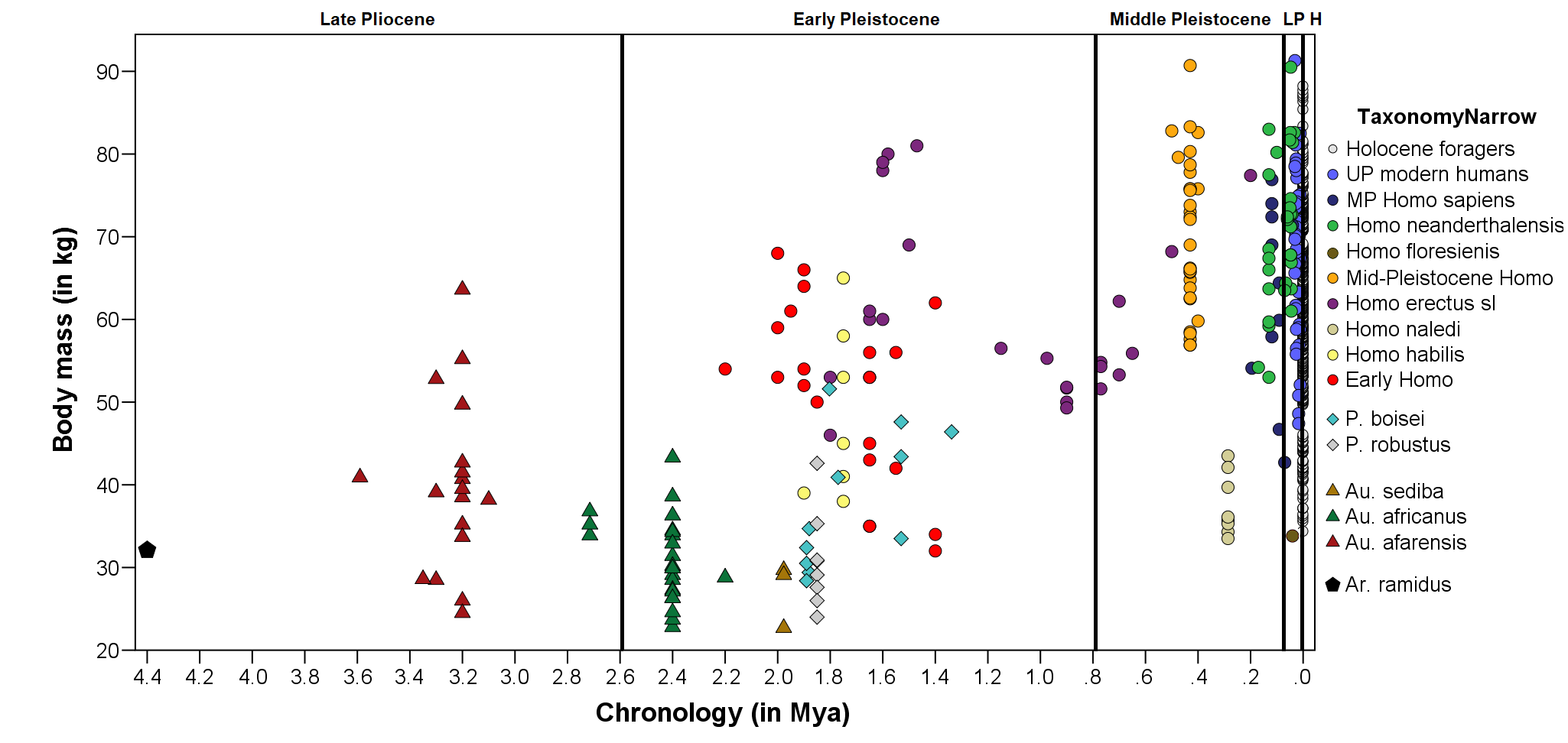


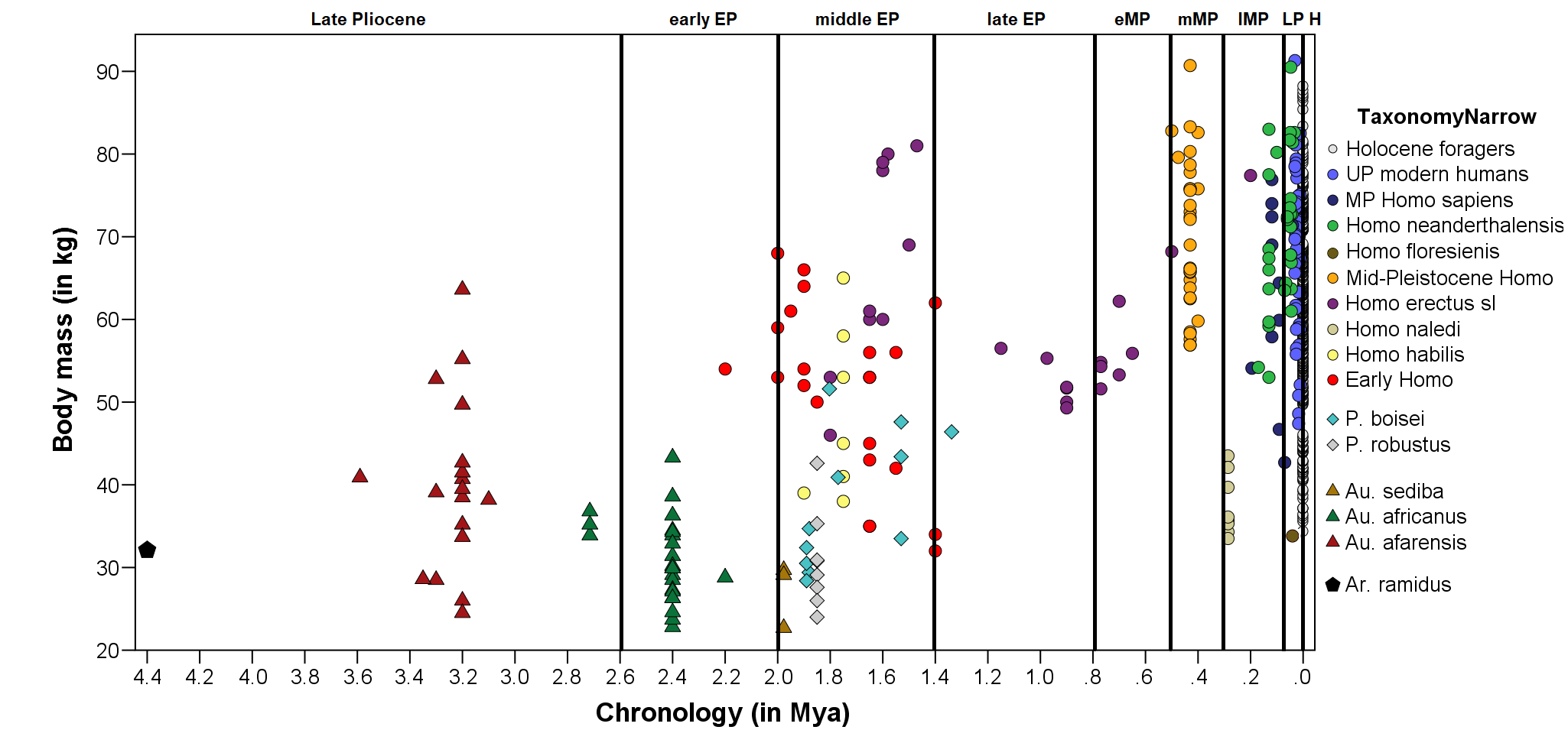


**Supplementary Figure 6. Stature estimates by time with temporal group boundaries.**

Stature estimates for all fossil hominins by time with indication of membership of narrow taxonomic group. *Top*: Coarse temporal groups; *bottom*: Fine temporal groups. Abbreviations: EP=Early Pleistocene; eMP=early Middle Pleistocene; mMP=middle Middle Pleistocene; lMP=late Middle Pleistocene; LP=Late Pleistocene; H=Holocene.

**
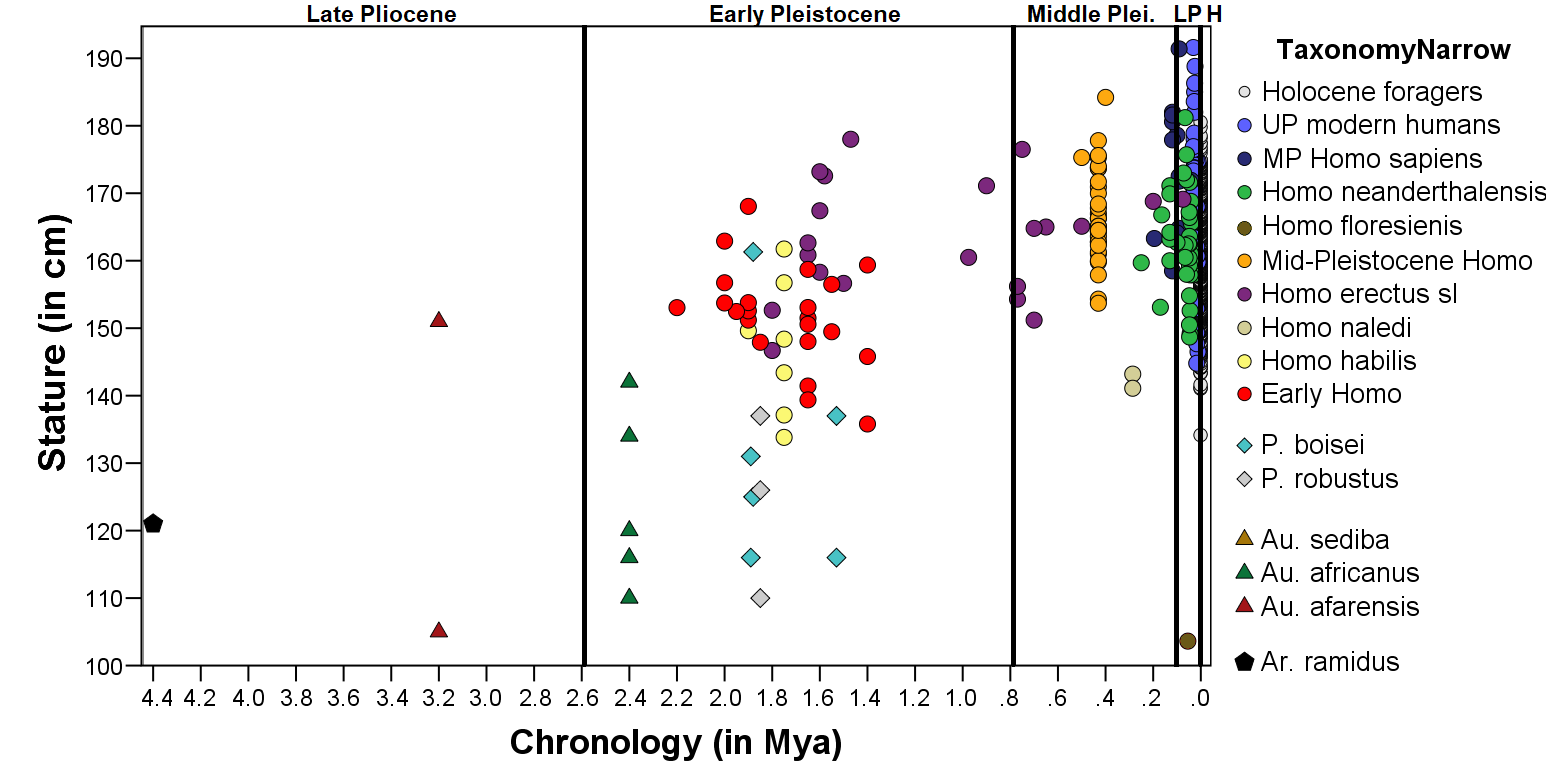
**

**
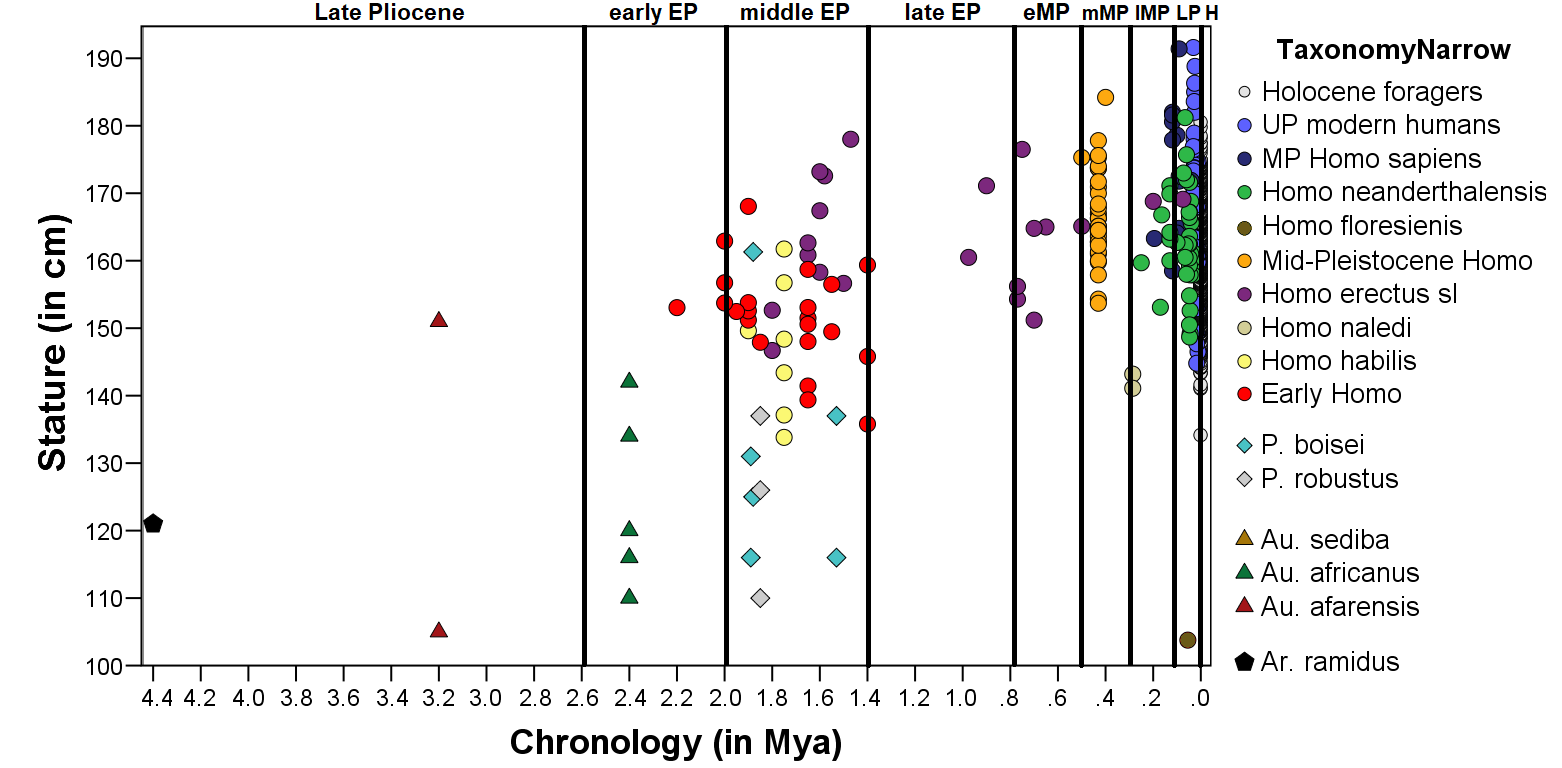
**

**Supplementary Figure 7. Within-lineage change during the Middle Pleistocene**

Scatter plot of body mass calculations for specimens of “Early Mid-Pleistocene *Homo*” and the SH hominins applying broad taxonomic categories (top). The graph shows a marked step increase in average mass after 0.5 Mya. In the narrow analyses, all labelled blue dots belong to the same group as the SH hominins (“Mid-Pleistocene *Homo*”; see plot bottom) while unlabelled blue dots are assigned to *Homo erectus* *s.l*. The plots support the notion of a step increase in body mass within Mid-Pleistocene *Homo* (SH hominins etc.) in comparison to late *Homo erectus*.

**
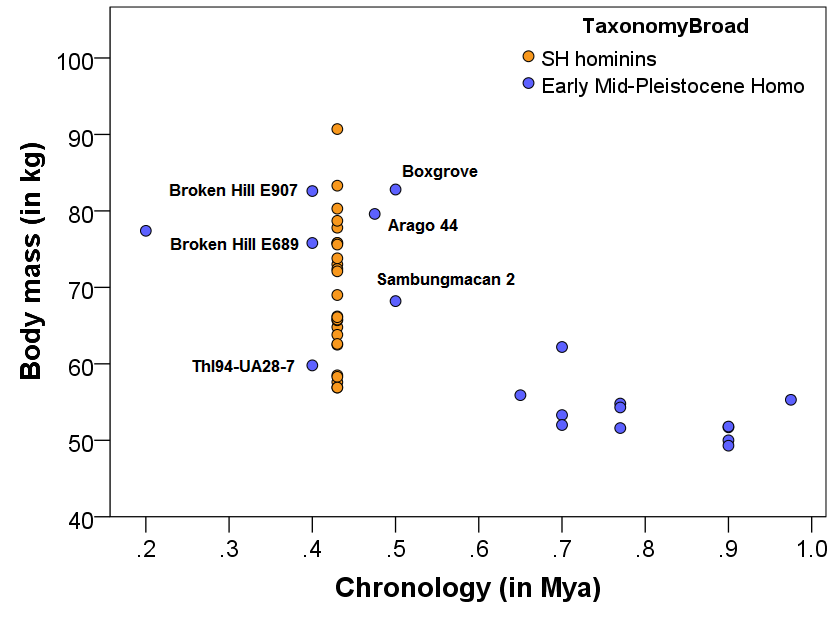
**

**
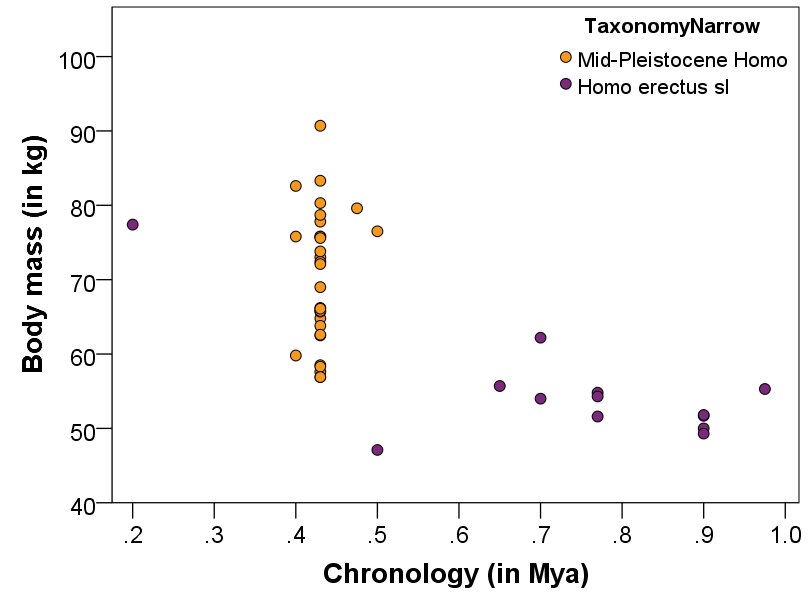
**

**Supplementary Figure 8. Within-lineage change in UP modern humans vs. Neanderthals**

Comparison of within-lineage chronological changes in body size between UP modern humans (top, blue) and Neanderthals (green, bottom). Note that while both body mass and stature are reduced significantly through time in UP modern humans, Neanderthals only show a significant increase in body mass while retaining similar stature (thereby also increasing their ponderal index through time).

**
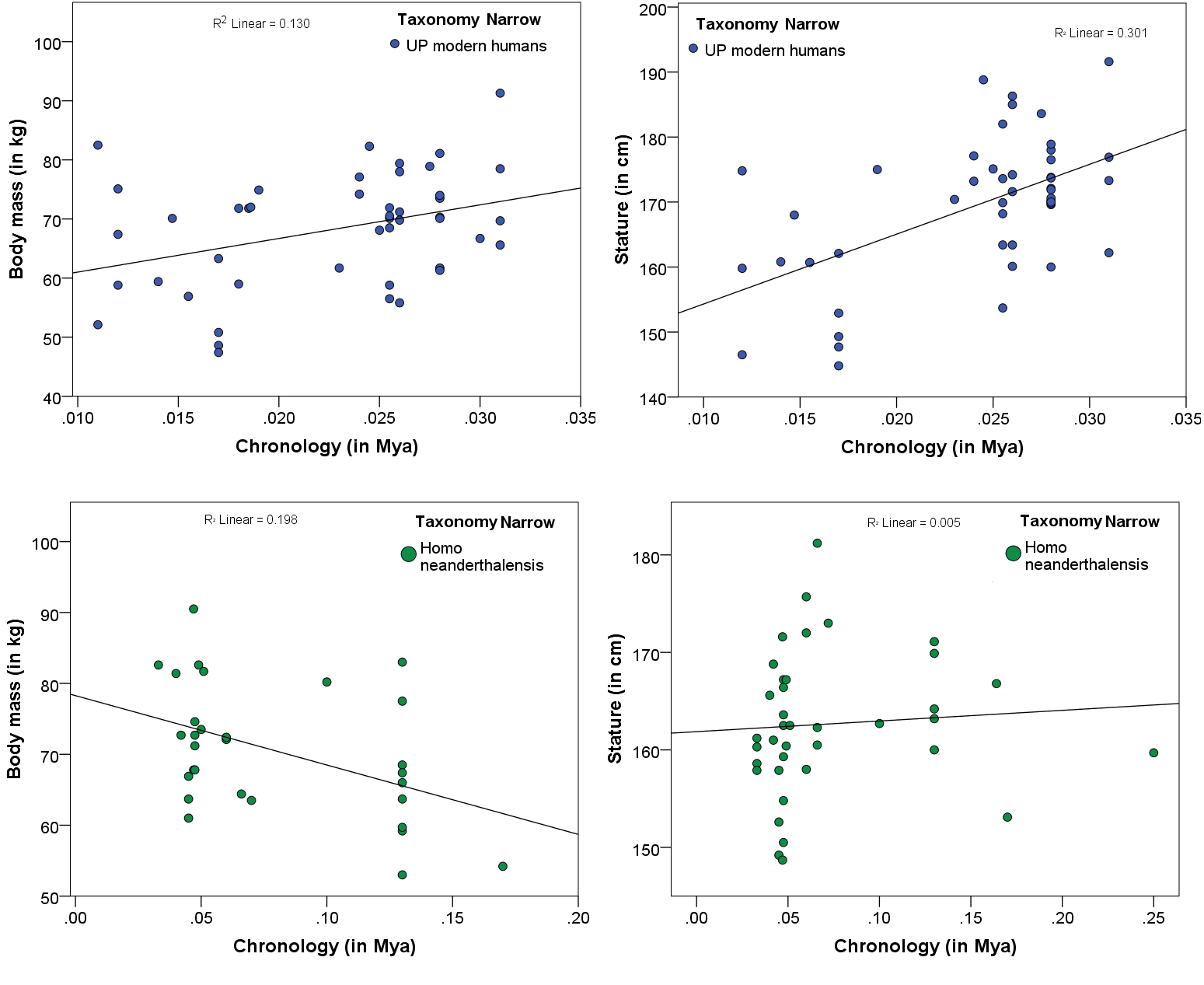
**

**Supplementary Figure 9. Body mass index scatter plot**

Scatter plot of BMI calculations for individual specimens by narrow taxonomic group

**
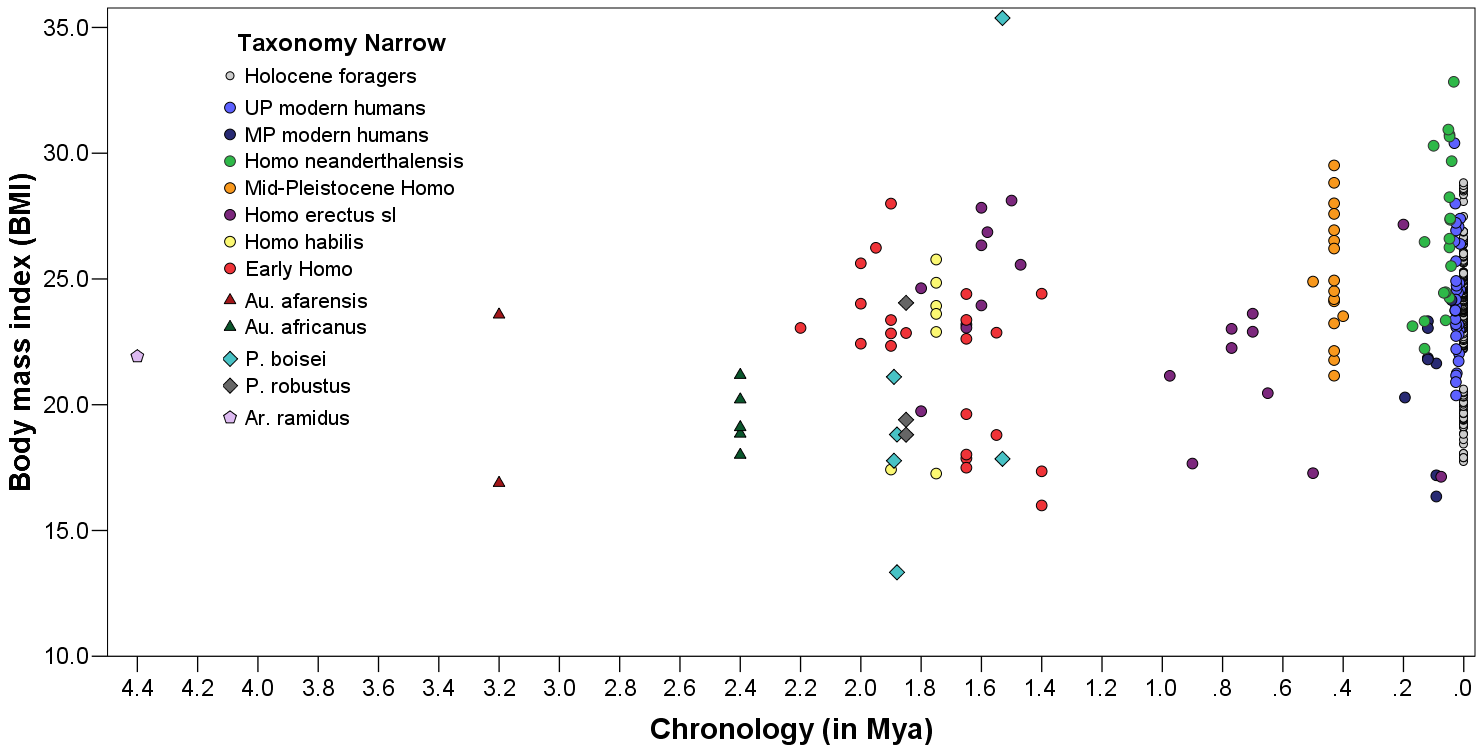
**

**Supplementary Tables**

**Supplementary Table 1. Comparison of body size estimates >1.0 Mya by key studies**

Body size estimates for the same fossils by different key studies. The focus is on the period >1.0 Mya as later estimates are generally considered to be more comparable. Estimates with %diff larger than 30% are removed for body mass (n=12) and %diff larger than 20% are removed for stature (n=1) to test the impact of these diverging estimates on the main results of this study (see Supplementary Text 2).

*Body mass*

| **Specimen** | **McHenry 1992** | **Grabowski et al. 2015** | **Will & Stock 2015** | **% diff MC/W&S^a^** | **%diff G/W&S^b^** | **%diff MC/G^c^** | **Removed in test?** |
| --- | --- | --- | --- | --- | --- | --- | --- |
| AL 211-1 | 59.7 | 52.8 |  |  |  | -11.6 |  |
| AL 288-1 | 27.9 | 26.0 |  |  |  | -6.9 |  |
| AL 333-3 | 50 | 38.5 |  |  |  | -22.9 |  |
| AL 333-95 | 62.9 | 49.7 |  |  |  | -21.0 |  |
| AL 333w-40 | 35 | 55.2 |  |  |  | 57.6 | Y |
| AL 333x-26 | 48.2 | 39.5 |  |  |  | -18.0 |  |
| D4167/3901 |  | 40.7 | 53 |  | 30.2 |  | Y |
| KNM-ER 1465 | 57.4 | 43.4 |  |  |  | -24.4 |  |
| KNM-ER 1471 | 39.1 | 37.2 |  |  |  | -4.9 |  |
| KNM-ER 1472 | 49.6 | 45.4 | 53 | 6.9 | 16.8 | -8.5 |  |
| KNM-ER 1473 | 62.5 |  | 68 | 8.8 |  |  |  |
| KNM-ER 1475 | 53.6 | 42.7 | 59 | 10.1 | 38.3 | -20.4 | Y |
| KNM-ER 1476 | 32.6 | 34.7 |  |  |  | 6.3 |  |
| KNM-ER 1481 | 57 | 40.9 | 61 | 7.0 | 49.2 | -28.3 | Y |
| KNM-ER 1500 | 42.2 | 28.4 |  |  |  | -32.6 | Y |
| KNM-ER 1503 | 39.7 | 30.5 |  |  |  | -23.1 |  |
| KNM-ER 164 | 51.4 |  | 60 | 16.7 |  |  |  |
| KNM-ER 1808 |  | 38.5 | 79 |  | 105.2 |  | Y |
| KNM-ER 1810 | 47.9 | 40.5 |  |  |  | -15.5 |  |
| KNM-ER 3228 | 61.6 | 50 | 66 | 7.1 | 32.0 | -18.8 | Y |
| KNM-ER 3728 | 45.2 |  | 64 | 41.6 |  |  | Y |
| KNM-ER 3735 | 37.4 | 38.4 | 39 | 4.3 | 1.6 | 2.7 |  |
| KNM-ER 5428 | 83.4 |  | 61 | -26.9 |  |  |  |
| KNM-ER 5880 | 55.9 | 45.1 |  |  |  | -19.3 |  |
| KNM-ER 5881 |  | 35.5 | 52 |  | 46.3 |  | Y |
| KNM-ER 736 |  | 65.5 | 80 |  | 22.1 |  |  |
| KNM-ER 737 | 70.9 | 64.1 | 78 | 10.0 | 21.6 | -9.6 |  |
| KNM-ER 738 | 37.2 | 29.4 |  |  |  | -20.9 |  |
| KNM-ER 741 | 47.6 |  | 60 | 26.1 |  |  |  |
| KNM-ER 803A | 67.1 | 54.8 | 69 | 2.8 | 26.0 | -18.4 |  |
| KNM-ER 813 | 51.5 |  | 50 | -2.9 |  |  |  |
| KNM-ER 815 | 41.6 | 40.9 |  |  |  | -1.8 |  |
| KNM-ER 993 | 61.3 | 33.5 |  |  |  | -45.3 | Y |
| KNM-WT 15000 (projected adult) |  | 64.4 | 80 |  | 24.2 |  |  |
| OH 20 | 52.4 | 51.6 |  |  |  | -1.6 |  |
| OH 35 |  | 35.5 | 45 |  | 26.7 |  |  |
| OH 53 | 49.3 |  | 53 | 7.5 |  |  |  |
| OH 62 | 33.2 | 27.3 | 38 | 14.5 | 39.4 | -17.9 | Y |
| OH 8 | 31.7 |  | 41 | 29.3 |  |  |  |
| SK 18b | 49 |  | 56 | 14.3 |  |  |  |
| SK 50 |  | 42.6 |  |  |  |  |  |
| SK 82 | 37.6 | 30.8 |  |  |  | -18.0 |  |
| SK 97 | 43 | 35.3 |  |  |  | -17.9 |  |
| Sts 14 | 30.3 | 22.8 |  |  |  | -24.9 |  |
| Stw 25 | 34.6 | 29.1 |  |  |  | -15.9 |  |
| Stw 311 | 40.8 | 33.9 | 42 | 2.9 | 23.9 | -16.9 |  |
| Stw 392 | 33 | 28.5 |  |  |  | -13.7 |  |
| Stw 431 | 61.6 | 34.5 |  |  |  | -44.0 | Y |
| Stw 443 | 41.4 | 34.3 |  |  |  | -17.1 |  |
| Stw 99 | 45.4 | 36.3 |  |  |  | -19.9 |  |

^a^ Percentual difference between the body mass estimate by McHenry (1992) and Will & Stock (2015)

^b^ Percentual difference between the body mass estimate by Grabowski (2015) and Will & Stock (2015)

^c^ Percentual difference between the body mass estimate by McHenry (1992) and Grabowski (2015)

*Stature*

| **Specimen** | **McHenry 1991** | **Ruff & Walker 1993** | **Lordkinapidze et al. 2007** | **Will & Stock 2015** | **% diff MC-R&W-L/W&S^a^** | **Removed in test?** |
| --- | --- | --- | --- | --- | --- | --- |
| KNM-ER 803A | 150 |  |  | 156.7 | 4.5 |  |
| KNM-ER 1472 | 150 |  |  | 153.7 | 2.5 |  |
| KNM-ER 1481 | 148 |  |  | 152.5 | 3.0 |  |
| KNM-ER 1808 | 181 | 173 |  | 173.19 | -4.3 / 0.1 |  |
| KNM-ER 3228 | 172 |  |  | 168.1 | -2.3 |  |
| KNM-ER 3728 | 142 |  |  | 151.2 | 6.5 |  |
| KNM-ER 736 | 180 | 180 |  | 172.6 | -4.1 |  |
| KNM-ER 737 | 157 | 160 |  | 167.4 | 6.6 / 4.6 |  |
| KNM-WT 15000 (projected adult) |  | 185 |  | 178 | -3.8 |  |
| OH 53 | 135 |  |  | 143.4 | 6.2 |  |
| OH 62 | 118 |  |  | 148.4 | **25.8** | Y |
| D2600 |  |  | 149.3 | 146.7 | -1.7 |  |
| D4111/D3442 |  |  | 143 | 152.7 | 6.8 |  |

^a^ Percentual difference between the stature estimates by McHenry (1992), Ruff & Walker (1993), Lordkipanidze *et al.* (2007) in comparison to Will & Stock (2015).

**Supplementary Table 2. Comparative test of body size estimates for Mid-Pleistocene *Homo*** (see also Supplementary Text 3 and Supplementary File 3)

**Table 2a.** Descriptives and comparisons of estimates deriving from different methods.

| **Group** | **In study** | | **Will & Stock prediction** | | | | **Comparison** | | | | | |
| --- | --- | --- | --- | --- | --- | --- | --- | --- | --- | --- | --- | --- |
|  | **Mean** | **CV** | | **Mean** | **CV** | | | **Diff .mean** | | | **Diff. CV** | |
| *Body mass* |  |  | |  |  |  | | |  | | | |
| middle Middle Pleistocene | 70.4 | 12.9 | | 66.3 | 15.1 | **-4.1** | | | **+2.2** | | | |
| SH hominins | 69.4 | 12.8 | | 64.8 | 12.3 | **-4.6** | | | **-0.5** | | | |
| Mid-Pleistocene *Homo* | 70.5 | 13.0 | | 66.5 | 13.4 | **-4.0** | | | | **+0.4** | | |
|  |  |  | |  |  |  | | |  | | |  |
| *Stature* |  |  | |  |  |  | | |  | | |  |
| middle Middle Pleistocene | 166.9 | 4.1 | | 161.9 | 3.2 | **-5.0** | | | **-0.9** | | | |
| SH hominins | 166.1 | 3.7 | | 161.4 | 3.1 | **-4.7** | | | **-0.6** | | | |
| Mid-Pleistocene *Homo* | 166.9 | 4.2 | | 161.8 | 3.2 | **-5.1** | | | **-1.0** | | | |

**Table 2b-1.** Comparison for fine chronological groups.

| **Body size** | **middle Middle**  **Pleistocene**  **(in study)** | **middle Middle**  **Pleistocene**  **(Will & Stock)** | **Early Middle**  **Pleistocene** | **Diff. (in study)** | **Diff.**  **(Will & Stock)** |
| --- | --- | --- | --- | --- | --- |
| Mean body mass | 70.4 | 66.3 | 54.9 | **+15.5** | **+11.4** |

**Table 2b-2.** Comparison for broad taxonomic groups.

| **Body size** | **SH hominins**  **(in study)** | **SH hominins**  **(Will & Stock)** | **Early Mid-Plei. *Homo*** | **Diff. (in study)** | **Diff.**  **(Will & Stock)** |
| --- | --- | --- | --- | --- | --- |
| Mean body mass | 69.4 | 64.8 | 61.5 | **+7.9** | **+3.3** |

**Table 2b-3.** Comparison for narrow taxonomic groups.

| **Body size** | **Mid-Plei. *Homo***  **(in study)** | **Mid-Plei. *Homo***  **(Will & Stock)** | ***Homo erectus* s.l.** | **Diff. (in study)** | **Diff.**  **(Will & Stock)** |
| --- | --- | --- | --- | --- | --- |
| Mean body mass | 70.5 | 66.5 | 60.8 | **+9.7** | **5.7** |

**Supplementary Table 3. Comparative test of body size estimates for early Homo vs. australopithecines** (see also Supplementary Text 3 and Supplementary File 3)

**Table 3a.** Descriptives and comparisons of estimates deriving from different methods.

| **Group** | **In study** | | **Will & Stock prediction** | | | | **Comparison** | | | | | |
| --- | --- | --- | --- | --- | --- | --- | --- | --- | --- | --- | --- | --- |
|  | **Mean** | **CV** | | **Mean** | **CV** | | | **Diff. mean** | | | **Diff. CV** | |
| Body mass |  |  | |  |  |  | | |  | | | |
| *Australopithecus* | 34.6 | 25.4 | | 39.2 | 27.4 | **+4.6** | | | **+2.0** | | | |
| *Paranthropus* | 35.0 | 22.0 | | 38.9 | 21.7 | **+3.9** | | | **-0.3** | | | |
| *P. boisei* | 38.1 | 21.5 | | 41.8 | 17.0 | **+3.7** | | | | **-4.5** | | |
| *P. robustus* | 31.7 | 19.4 | | 36.3 | 25.9 | **+4.6** | | | | **+6.5** | | |
| *Au. sediba* | 27.2 | 14.3 | | 30.0 | 21.1 | **+2.8** | | | | **+6.8** | | |
| *Au. africanus* | 31.1 | 16.2 | | 35.1 | 21.3 | **+4.0** | | | | **+5.1** | | |
| *Au. afarensis* | 39.9 | 25.9 | | 45.2 | 25.2 | **+5.3** | | | | **-0.7** | | |
|  |  |  | |  |  |  | | | |  | | |
| Stature |  |  | |  |  |  | | |  | | |  |
| *Australopithecus* | 125.4 | 13.7 | | 136.1 | 8.9 | **+10.7** | | | **-4.8** | | | |
| *Paranthropus* | 128.8 | 12.0 | | 137.0 | 5.6 | **+8.2** | | | **-6.4** | | | |
| *P. boisei* | 131.1 | 13.0 | | 137.7 | 5.4 | **+6.6** | | | **-7.6** | | | |
| *P. robustus* | 124.3 | 10.9 | | 135.6 | 7.1 | **+11.3** | | | **-3.8** | | | |
| *Au. africanus* | 124.4 | 10.6 | | 135.6 | 6.9 | **+11.2** | | | **-3.7** | | | |
| *Au. afarensis* | 128.0 | 25.4 | | 137.6 | 16.8 | **+9.6** | | | **-8.6** | | | |

**Table 3b-1.** Comparison for broad taxonomic groups (*Australopithecus*).

| **Body size** | ***Australopithecus***  **(in study)** | ***Australopithecus***  **(Will & Stock)** | **Early *Homo***  **(without erectus)** | **Diff. (in study)** | **Diff.**  **(Will & Stock)** |
| --- | --- | --- | --- | --- | --- |
| Mean body mass | 34.6 | 39.2 | 50.6 | **+16.0** | **+12.4** |
| Mean stature | 125.4 | 136.1 | 150.8 | **+25.4** | **+14.7** |

**Table 3b-2.** Comparison for broad taxonomic groups (*Paranthropus*).

| **Body size** | ***Paranthropus***  **(in study)** | ***Paranthropus***  **(Will & Stock)** | **Early *Homo***  **(without erectus)** | **Diff. (in study)** | **Diff.**  **(Will & Stock)** |
| --- | --- | --- | --- | --- | --- |
| Mean body mass | 35.0 | 38.9 | 50.6 | **+15.6** | **+11.7** |
| Mean stature | 128.8 | 137.0 | 150.8 | **+22.0** | **+13.8** |

**Supplementary Table 4. Control for size estimates by body parts for analytical categories**.

Tables 4a-4e provide a general overview (see Supplementary File 1 for data by specimen), showing that lower limb elements make up the majority for body size predictions in all groups, mostly >90%. Tables 4f-4g compare mean values and CVs for estimates from lower limbs only vs. all estimates for the few groups which show a higher representation of upper limb and axial elements (>10%) to test for the effect of excluding the latter body parts for body size predictions. The results show that differences in means are negligible for all temporal groups (0.1-1.8%) and for most taxonomic groups (stature: 0.1-1.6% body mass: 4.2-10.7%), but never reach >11% differences. CVs show minor variation for temporal groups (3.2-11.9%), with some higher differences in taxonomic groups (3.1-45.2%), particularly for those with small sample sizes. The latter do, however, not impact main results of this study with regard to patterns of relative differences or statistical significance.

**Table 4a.** Overview of body part representation for body size estimates for the entire hominin sample.

| **Overall** | **Lower limb** | **Upper limb** | **Lower & Upper limb** | **Axial** |
| --- | --- | --- | --- | --- |
| Body size | 241 | 11 | - | 2 |
| Stature | 136 | 28 | 38 | 2 |

**Table 4b.** Body part representation for body mass estimates by temporal groups. Only the Early Pleistocene (13.3%), early Middle Pleistocene (14.3%) and middle Early Pleistocene (20.3%) groups show higher amounts of estimates from the upper limbs and axial skeleton. Lower limb elements still make up the large majority of these groups though (>79%).

| **Coarse temporal group** | **Lower limb** | **Upper limb** | **Axial** | **Fine temporal group** | **Lower limb** | **Upper limb** | **Axial** |
| --- | --- | --- | --- | --- | --- | --- | --- |
| Late Pleistocene | 78 | - | - | Late Pleistocene | 78 | - | - |
| Middle Pleistocene | 59 | 1 | - | late Middle Pleistocene | 21 | - | - |
| Early Pleistocene | 78 | 10 | 2 | middle Middle Pleistocene | 32 | - | - |
| Late Pliocene | 26 | - | - | early Middle Pleistocene | 6 | 1 | - |
|  |  |  |  | late Early Pleistocene | 8 | - | - |
|  |  |  |  | middle Early Pleistocene | 47 | 10 | 2 |
|  |  |  |  | early Early Pleistocene | 23 | - | - |
|  |  |  |  | Late Pliocene | 26 | - | - |

**Table 4c.** Body part representation for stature estimates by temporal groups. Only the Early Pleistocene (21.4%), Late Pleistocene (15.2%), early, middle and late Middle Pleistocene (11.1-33%) groups show higher amounts of estimates from the upper limbs only and axial skeleton. Lower limb elements still make up the large majority of these groups though (>66%).

| **Coarse temporal group** | **Lower limb** | **Lower & Upper** | **Upper limb** | **Axial** | **Fine temporal group** | **Lower limb** | **Lower & Upper** | **Upper limb** | **Axial** |
| --- | --- | --- | --- | --- | --- | --- | --- | --- | --- |
| Late Pleistocene | 41 | 37 | 14 | - | Late Pleistocene | 41 | 37 | 14 | - |
| Middle Pleistocene | 49 | 3 | 2 | - | late Middle Pleistocene | 8 | 2 | 2 | - |
| Early Pleistocene | 44 | 2 | 10 | - | middle Middle Pleistocene | 33 | - | - | - |
| Late Pliocene | 3 | - | - | - | early Middle Pleistocene | 8 | - | 1 | - |
|  |  |  |  |  | late Early Pleistocene | 3 | - | - | - |
|  |  |  |  |  | middle Early Pleistocene | 35 | 2 | 10 | - |
|  |  |  |  |  | early Early Pleistocene | 6 | - | - | - |
|  |  |  |  |  | Late Pliocene | 3 | - | - | - |

**Table 4d.** Body part representation for body mass estimates by taxonomic groups. Only early *Homo* (30.8%) in the broad category early *Homo* (40.9%) and *Homo habilis* (28.6%) in the narrow category show higher amounts of estimates from the upper limbs and axial skeleton. Lower limb elements still make up the majority of these groups.

| **Broad taxonomic group** | **Lower limb** | **Upper limb** | **Axial** | **Narrow taxonomic group** | **Lower limb** | **Upper limb** | **Axial** |
| --- | --- | --- | --- | --- | --- | --- | --- |
| Pleistocene *Homo sapiens*^b^ | 57 | - | - | UP modern humans | 46 | - | - |
| Neanderthals | 31 | - | - | MP *Homo sapiens* | 11 | - | - |
| SH hominins | 26 | - | *-* | *Homo neanderthalensis* | 31 | - | - |
| Early Mid-Pleistocene *Homo* | 20 | 1 | - | Mid-Pleistocene *Homo*^c^ | 31 | - | - |
| Homo naledi | 8 | - | - | *Homo erectus sl* | 23 | 1 | 1 |
| Early *Homo* | 28 | 10 | 2 | Homo naledi | 8 | - | - |
| Australopithecines | 47 | - | - | Early *Homo* | 13 | 8 | 1 |
| Paranthropines | 20 | - | *-* | *Homo habilis* | 5 | 2 | - |
| *Ardipithecus* | 1 | - | *-* | *Au. afarensis* | 18 | - | - |
|  |  |  |  | *Au. africanus* | 24 | - | - |
|  |  |  |  | *Au. sediba* | 3 | - | - |
|  |  |  |  | *P. boisei* | 11 | - | - |
|  |  |  |  | *P. robustus* | 9 | - | - |
|  |  |  |  | *Ar. ramidus* | 1 | - | - |

**Table 4e.** Body part representation for stature estimates by taxonomic groups. Pleistocene *Homo sapiens* (11.7%), Neanderthals (21.1%) and early *Homo* (30.8%) in the broad category, as well as UP modern humans (10.7%), *Homo neanderthalensis* (21.1%), early *Homo* (40.9%), *Homo habilis* (28.6%) and *Homo erectus sl*. (14.3%) in the narrow category show higher amounts of estimates from the upper limbs only and axial skeleton. Lower limb elements still make up the majority of these groups. *Homo naledi* shows 50% upper limb estimates by a sample size of only n=2.

| **Broad taxonomic group** | **Lower limb** | **Upper & Lower** | **Upper limb** | **Axial** | **Narrow taxonomic group** | **Lower limb** | **Upper & Lower** | **Upper limb** | **Axial** |
| --- | --- | --- | --- | --- | --- | --- | --- | --- | --- |
| Pleistocene *Homo sapiens*^b^ | 24 | 29 | 7 | - | UP modern humans | 26 | 24 | 6 | - |
| Neanderthals | 21 | 9 | 8 | - | MP *Homo sapiens* | 7 | 5 | 1 | - |
| SH hominins | 30 | *-* | - | *-* | *Homo neanderthalensis* | 21 | 9 | 8 | - |
| Early Mid-Pleistocene *Homo* | 12 | - | 2 | - | Mid-Pleistocene *Homo*^c^ | 32 | - | - | - |
| *Homo naledi* | 1 | - | 1 | - | *Homo erectus sl* | 18 | - | 2 | 1 |
| Early *Homo* | 27 | - | 10 | 2 | *Homo naledi* | 1 | - | 1 | - |
| Australopithecines | 7 | - | - | - | Early *Homo* | 13 | - | 8 | 1 |
| Paranthropines | 5 | *-* | - | *-* | *Homo habilis* | 5 | - | 2 | - |
| *Ardipithecus* | 1 | *-* | - | *-* | *Au. afarensis* | 2 | - | - | - |
|  |  |  |  |  | *Au. africanus* | 5 | - | - | - |
|  |  |  |  |  | *Au. sediba* | - | - | - | - |
|  |  |  |  |  | *P. boisei* | 6 | - | - | - |
|  |  |  |  |  | *P. robustus* | 3 | - | - | - |
|  |  |  |  |  | *Ar. ramidus* | 1 | - | - | - |

**Table 4f.** Comparison of means and CVs for body mass and stature between all estimates (in the study) and estimates from lower limbs only by selected temporal groups.

| **Temporal group** | **Mean**  **(in study)** | **CV**  **(in study)** | **Mean (lower limbs only)** | **CV (lower limbs only)** | **%diff mean** | **%diff CV** |
| --- | --- | --- | --- | --- | --- | --- |
| *Body mass* |  |  |  |  |  |  |
| Early Pleistocene | 43.8 | 34.5 | 43.0 | 35.6 | **-1.8** | **+3.2** |
| early Middle Pleistocene | 54.9 | 6.5 | 54.7 | 7.1 | **-0.4** | **+9.2** |
| middle Early Pleistocene | 47.1 | 32.7 | 46.5 | 34.4 | **-1.3** | **+5.2** |
| *Stature* |  |  |  |  |  |  |
| Late Pleistocene | 167.4 | 6.7 | 167.2 | 7.5 | **-0.1** | **+11.9** |
| Early Pleistocene | 148.0 | 11.3 | 147.0 | 12.3 | **-0.7** | **+8.8** |
| early Middle Pleistocene | 159.7 | 6.0 | 158.8 | 6.5 | **-0.6** | **+8.3** |
| middle Early Pleistocene | 149.1 | 10.0 | 148.2 | 11.1 | **-0.6** | **+11.0** |

**Table 4g.** Comparison of means and CVs for body mass and stature between all estimates (in the study) and estimates from lower limbs only by selected taxonomic groups.

| **Taxonomic group** | **Mean** | **CV** | **Mean (lower limbs only)** | **CV (lower limbs only)** | **%diff mean** | **%diff CV** |
| --- | --- | --- | --- | --- | --- | --- |
| *Body mass* |  |  |  |  |  |  |
| Early *Homo* (coarse) | 54.7 | 23.5 | 57.0 | 21.2 | **+4.2** | **-9.8** |
| Early *Homo* (narrow) | 51.2 | 20.8 | 55.2 | 11.4 | **+7.8** | **-45.2** |
| *Homo habilis* | 48.4 | 21.5 | 43.2 | 14.1 | **-10.7** | **-34.4** |
| *Stature* |  |  |  |  |  |  |
| Pleistocene *Homo sapiens* | 170.3 | 5.2 | 170.5 | 6.0 | **+0.1** | **+15.4** |
| Neanderthals | 162.7 | 4.5 | 162.1 | 4.8 | **-0.4** | **+6.7** |
| Early *Homo* (broad) | 153.9 | 6.5 | 154.8 | 6.7 | **+0.6** | **+3.1** |
| UP modern humans | 169.4 | 6.4 | 169.5 | 6.0 | **+0.1** | **-6.3** |
| *Homo erectus sl* | 163.4 | 5.2 | 162.1 | 5.9 | **-0.8** | **+13.5** |
| *Homo naledi* | 142.2 | 1.0 | 143.2 | - | **+1.1** | **-** |
| Early *Homo* (narrow) | 151.9 | 4.9 | 153.8 | 3.2 | **+1.3** | **-34.7** |
| *Homo habilis* | 147.3 | 6.8 | 144.9 | 5.9 | **-1.6** | **-13.2** |

**Supplementary Table 5. Temporal groupings of body size estimates**

Coarse and fine time groups used for analysing temporal trends of body size estimates in the hominin sample. See Supplementary File 1 for the attribution of individual specimens to groups.

| **Coarse temporal group** | **Timeframe (in Mya)** | **n**  **(body mass)** | **n (stature)** | **Fine temporal group** | **Timeframe**  **(in Mya)** | **n**  **(body mass)** | **n**  **(stature)** |
| --- | --- | --- | --- | --- | --- | --- | --- |
| Holocene^a^ | 0.010-0.001 | 438 | 449 | Holocene^a^ | 0.010-0.001 | 438 | 449 |
| Late Pleistocene | 0.125-0.011 | 78 | 92 | Late Pleistocene | 0.125-0.011 | 78 | 92 |
| Middle Pleistocene | 0.78-0.126 | 60 | 52 | late Middle Pleistocene | 0.30-0.126 | 21 | 12 |
| Early Pleistocene | 2.58-0.781 | 90 | 56 | middle Middle Pleistocene | 0.50-0.30 | 32 | 33 |
| Late Pliocene | 4.40-2.58 | 24 | 3 | early Middle Pleistocene | 0.78-0.501 | 7 | 7 |
|  |  |  |  | late Early Pleistocene | 1.39-0.781 | 8 | 3 |
|  |  |  |  | middle Early Pleistocene | 2.00-1.40 | 59 | 47 |
|  |  |  |  | early Early Pleistocene | 2.58-2.01 | 23 | 6 |
|  |  |  |  | Late Pliocene | 4.40-2.58 | 24 | 3 |

^a^The Holocene forager sample is provided as comparative baseline.

**Supplementary Table 6. Taxonomic groupings of body size estimates**

Groupings used for analysing taxonomic trends of body size estimates in the hominin sample, ordered through time from younger to older. See Supplementary File 1 for the attribution of individual specimens to groups.

| **Broad taxonomic group** | **n**  **(body mass)** | **n (stature)** | **Narrow taxonomic group** | **n**  **(body mass)** | **n (stature)** |
| --- | --- | --- | --- | --- | --- |
| Holocene foragers^a^ | 438 | 449 | Holocene foragers^a^ | 438 | 449 |
| Pleistocene *Homo sapiens*^b^ | 57 | 60 | UP modern humans | 46 | 47 |
| Neanderthals | 30 | 38 | MP *Homo sapiens* | 11 | 13 |
| SH hominins | 26 | 31 | *Homo neanderthalensis* | 30 | 38 |
| Early Mid-Pleistocene *Homo* | 19 | 13 | Mid-Pleistocene *Homo*^c^ | 31 | 32 |
| *Homo naledi* | 8 | 2 | *Homo erectus sl* | 24 | 21 |
| Early *Homo* | 40 | 39 | *Homo naledi* | 8 | 2 |
| Australopithecines | 47 | 7 | Early *Homo* | 22 | 22 |
| Paranthropines | 21 | 9 | *Homo habilis* | 7 | 7 |
| *Ardipithecus* | 1 | 1 | *Au. afarensis* | 18 | 2 |
|  |  |  | *Au. africanus* | 24 | 5 |
|  |  |  | *Au. sediba* | 3 | - |
|  |  |  | *P. boisei* | 11 | 6 |
|  |  |  | *P. robustus* | 9 | 3 |
|  |  |  | *Ar. ramidus* | 1 | 1 |

^a^ The Holocene forager sample is provided as comparative baseline.

^b^ Combined data from MP and UP modern humans. The Holocene forager sample is excluded.

^c^ We use the term here as a shorthand to denote all African and European Middle Pleistocene hominins that predate Neanderthals and are not *Homo erectus s.l.* or *Homo naledi*. While this group could be called *Homo heidelbergensis*, including *Homo rhodesiensis* as the African representatives of this lineage (e.g., Mounier et al. 2009; Stringer 2012), Bermúdez de Castro *et al.* (2004a) and Arsuaga *et al.* (2014) reject naming the SH hominins as *Homo heidelbergensis* which make up the majority of this group.

**Supplementary Table 7. Regression of body size and time with different types of fit**

Body mass

Model summary and parameter estimates. Dependent variable: body mass; Independent variable: Chronology in Mya.

| **Equation** | **Model Summary** | | | | | | | **Parameter Estimates** | | | | | | | |  |
| --- | --- | --- | --- | --- | --- | --- | --- | --- | --- | --- | --- | --- | --- | --- | --- | --- |
|  | **R²** | **F** | **df1** | | **df2** | | **Sig.** | **Constant** | | | **b1** | | **b2** | | **b3** |  |
| Linear | .475 | 226.551 | 1 | 250 | | .000 | | | 67.714 | -11.213 | |  | |  | |  |
| Quadratic | .497 | 123.209 | 2 | 249 | | .000 | | | 69.654 | -18.254 | | 2.407 | |  | |  |
| Cubic | .506 | 84.750 | 3 | 248 | | .000 | | | 68.309 | -8.178 | | -5.233 | | 1.402 | |  |
| Power | .398 | 165.142 | 1 | 250 | | .000 | | | 46.590 | -.122 | |  | |  | |  |
| Growth | .480 | 231.156 | 1 | 250 | | .000 | | | 4.204 | -.226 | |  | |  | |  |
| Exponential | .480 | 231.156 | 1 | 250 | | .000 | | | 66.951 | -.226 | |  | |  | |  |
| The independent variable is Chronology. | | | | | | | | | | | | | | | | |

Stature

Model summary and parameter estimates. Dependent variable: stature; Independent variable: Chronology in Mya.

|  |  |  |  |  |  |  |  |  |  |  |
| --- | --- | --- | --- | --- | --- | --- | --- | --- | --- | --- |
| **Equation** | **Model Summary** | | | | | **Parameter Estimates** | | | | |
|  | **R ²** | **F** | **df1** | **df2** | **Sig.** | **Constant** | **b1** | **b2** | **b3** |  |
| Linear | .475 | 226.551 | 1 | 250 | .000 | 67.714 | -11.213 |  |  |  |
| Quadratic | .497 | 123.209 | 2 | 249 | .000 | 69.654 | -18.254 | 2.407 |  |  |
| Cubic | .506 | 84.750 | 3 | 248 | .000 | 68.309 | -8.178 | -5.233 | 1.402 |  |
| Power | .398 | 165.142 | 1 | 250 | .000 | 46.590 | -.122 |  |  |  |
| Growth | .480 | 231.156 | 1 | 250 | .000 | 4.204 | -.226 |  |  |  |
| Exponential | .480 | 231.156 | 1 | 250 | .000 | 66.951 | -.226 |  |  |  |

**Supplementary Table 8. Linear regressions for within-lineage temporal change**

Body mass

Linear regressions for broad and taxonomic groupings with n>15. Dependent variable: Body mass; independent variable: Time (in Mya). Only those groupings are included which have body size estimates over a certain time trange.

| **Taxonomic grouping** | **n** | **Slope** | ***F*** | ***p*** | **Time range**  **(in Mya)** |
| --- | --- | --- | --- | --- | --- |
| Broad |  |  |  |  |  |
| Pleistocene *Homo sapiens* | 57 | -0.143 | 1.147 | 0.289 | 0.011-0.195 |
| Neanderthals | 30 | **-0.445** | 6.909 | **0.014** | 0.033-0.170 |
| *SH hominins* | 26 | - | - | - |  |
| Early Mid-Pleistocene *Homo* | 19 | **-0.826** | 36.379 | <**0.001** | 0.20-0.975 |
| Early *Homo* | 40 | 0.001 | 0.001 | 0.999 | 1.15-2.20 |
| Australopithecines | 47 | **0.559** | 20.422 | **<0.001** | 1.98-4.10 |
| Paranthropines | 20 | -0.445 | 4.45 | 0.049 | 1.34-2.66 |
|  |  |  |  |  |  |
| Narrow |  |  |  |  |  |
| UP modern humans | 46 | **0.361** | 6.594 | **0.014** | 0.011-0.031 |
| Neanderthals | 30 | **-0.445** | 6.909 | **0.014** | 0.033-0.170 |
| Mid-Pleistocene Homo | 31 | 0.217 | 1.431 | 0.241 | 0.500-0.400 |
| Homo erectus s.l. | 24 | 0.186 | 0.791 | 0.383 | 0.20-1.80 |
| Early *Homo* | 22 | **0.561** | 9.168 | **0.007** | 1.40-2.20 |
| *Au. afarensis* | 18 | -0.060 | 0.057 | 0.814 | 3.10-3.60 |
| *Au. africanus* | 24 | 0.329 | 2.661 | 0.117 | 2.20-2.71 |
|  |  |  |  |  |  |
| Combined taxonomies |  |  |  |  |  |
| Mid-Pleistocene Homo  & Neanderthals | 61 | -0.042 | 0.102 | 0.751 | 0.033-0.500 |
|  |  |  |  |  |  |

Significant regressions in **boldface**

**Red slope**: Declining body mass over time

**Green slope**: Increasing body mass over time

Stature

Linear regressions for broad and taxonomic groupings with n>5. Dependent variable: Stature; independent variable: Time (in Mya).

**Stature**

| **Taxonomic grouping** | **n** | **Slope** | ***F*** | ***p*** | **Time range**  **(in Mya)** |
| --- | --- | --- | --- | --- | --- |
| Broad |  |  |  |  |  |
| Pleistocene *Homo sapiens* | 60 | 0.189 | 2.148 | 0.148 | 0.011-0.195 |
| Neanderthals | 38 | 0.073 | 0.192 | 0.664 | 0.033-0.250 |
| *SH hominins* | - | - | - | - |  |
| Early Mid-Pleistocene *Homo* | 13 | -0.396 | 2.047 | 0.180 | 0.20-0.975 |
| Early *Homo* | 39 | -0.065 | 0.158 | 0.694 | 1.40-2.20 |
| Australopithecines | 7 | 0.102 | 0.054 | 0.828 | 2.40-3.20 |
| Paranthropines | 9 | 0.103 | 0.075 | 0.792 | 1.53-1.89 |
|  |  |  |  |  |  |
| Narrow |  |  |  |  |  |
| UP modern humans | 47 | **0.549** | 19.739 | **<0.001** | 0.011-0.031 |
| MP *Homo sapiens* | 13 | -0.185 | 0.388 | 0.546 | 0.004-0.250 |
| Neanderthals | 38 | 0.073 | 0.192 | 0.664 | 0.033-0.200 |
| Mid-Pleistocene Homo | 32 | 0.023 | 0.016 | 0.899 | 0.500-0.400 |
| Homo erectus s.l. | 21 | -0.194 | 0.746 | 0.399 | 0.20-1.80 |
| Early *Homo* | 22 | 0.409 | 4.017 | 0.059 | 1.40-2.20 |
|  |  |  |  |  |  |
| Combined taxonomies |  |  |  |  |  |
| Mid-Pleistocene Homo  & Neanderthals | 70 | **0.294** | 6.418 | **0.014** | 0.033-0.500 |
|  |  |  |  |  |  |

Significant regressions in **boldface**

**Red slope**: Declining stature over time

**Supplementary Table 9. Ponderal index^1^ for temporal groups**

a) Summary statistics of ponderal index by coarse temporal group.

| **Coarse temporal group** | **n** | **Mean** | **Median** | **SD** | **Range** | **CV** |
| --- | --- | --- | --- | --- | --- | --- |
| Holocene | 438 | 15.08 | 15.29 | 0.675 | 4.10 | 4.48 |
| Late Pleistocene | 49 | 14.98 | 14.71 | 2.459 | 12.16 | 16.42 |
| Middle Pleistocene | 29 | 14.68 | 14.98 | 1.573 | 6.58 | 10.72 |
| Early Pleistocene | 56 | 15.06 | 14.95 | 3.133 | 22.23 | 20.80 |
| Late Pliocene | 3 | 17.25 | 18.12 | 5.688 | 11.28 | 32.97 |

b) Summary statistics of ponderal index by fine temporal group.

| **Fine temporal group** | **n** | **Mean** | **Median** | **SD** | **Range** | **CV** |
| --- | --- | --- | --- | --- | --- | --- |
| Holocene | 438 | 15.08 | 15.29 | 0.675 | 4.10 | 4.48 |
| Late Pleistocene | 59 | 14.98 | 14.71 | 2.516 | 12.16 | 16.42 |
| late Middle Pleistocene | 6 | 14.54 | 14.84 | 1.336 | 3.67 | 9.19 |
| middle Middle Pleistocene | 17 | 14.80 | 15.24 | 1.798 | 6.58 | 12.15 |
| early Middle Pleistocene | 6 | 14.49 | 14.58 | 1.273 | 3.45 | 8.79 |
| late Early Pleistocene | 3 | 12.57 | 13.08 | 2.039 | 3.98 | 16.22 |
| middle Early Pleistocene | 47 | 15.14 | 15.05 | 3.259 | 22.23 | 21.53 |
| early Early Pleistocene | 6 | 15.70 | 15.95 | 2.074 | 5.58 | 13.21 |
| Late Pliocene | 3 | 17.25 | 18.12 | 5.688 | 11.28 | 32.97 |

^1^ Ponderal Index=mass/stature³.

**Supplementary Table 10. Ponderal index for taxonomic groups.**

a) Summary statistics of ponderal index by broad taxonomic group.

| **Taxonomic grouping** | **n** | **Mean** | **Median** | **SD** | **Range** | **CV** |
| --- | --- | --- | --- | --- | --- | --- |
| Holocene foragers^a^ | 438 | 15.08 | 15.29 | 0.675 | 4.10 | 4.48 |
| Pleistocene *Homo sapiens^b^* | 42 | 14.05 | 14.02 | 2.041 | 10.16 | 14.53 |
| Neanderthals | 20 | 16.58 | 16.02 | 2.233 | 7.41 | 13.47 |
| SH hominins | 15 | 15.13 | 15.32 | 1.486 | 4.51 | 9.82 |
| Early Mid-Pleistocene *Homo* | 11 | 13.74 | 14.20 | 2.002 | 5.77 | 14.57 |
| Early *Homo* | 39 | 14.80 | 15.06 | 1.904 | 7.55 | 12.86 |
| Australopithecines | 7 | 16.12 | 16.84 | 3.786 | 11.28 | 23.49 |
| Paranthropines | 9 | 16.62 | 15.05 | 6.391 | 22.23 | 38.45 |
| Ardipithecus | 1 | 18.12 | 18.12 | - | - |  |

b) Summary statistics of ponderal index by narrow taxonomic group.

| **Taxonomic grouping** | **n** | **Mean** | **Median** | **SD** | **Range** | **CV** |
| --- | --- | --- | --- | --- | --- | --- |
| Holocene foragers | 438 | 15.08 | 15.29 | .675 | 4.10 | 4.48 |
| UP modern humans | 33 | 14.56 | 14.28 | 1.812 | 7.07 | 12.45 |
| MP *Homo sapiens* | 9 | 12.18 | 12.42 | 1.804 | 6.00 | 14.81 |
| *Homo neanderthalensis* | 20 | 16.58 | 16.01 | 2.233 | 7.41 | 13.47 |
| Mid-Pleistocene *Homo* | 16 | 15.07 | 15.28 | 1.455 | 4.51 | 9.65 |
| *Homo erectus sl* | 19 | 14.43 | 14.42 | 1.983 | 7.82 | 13.74 |
| Early *Homo* | 22 | 14.44 | 14.71 | 1.824 | 7.55 | 12.63 |
| *Homo habilis* | 7 | 15.18 | 15.37 | 2.639 | 6.34 | 17.38 |
| *P. boisei* | 6 | 16.43 | 14.31 | 7.605 | 22.23 | 46.29 |
| *P. robustus* | 3 | 17.00 | 15.40 | 4.296 | 8.14 | 25.27 |
| *Au. africanus* | 5 | 15.83 | 16.84 | 2.292 | 5.58 | 14.48 |
| *Au. afarensis* | 2 | 16.82 | 16.82 | 7.974 | 11.28 | 47.41 |
| *Ardipithecus* | 1 | 18.12 | 18.12 | - | - |  |

**Supplementary Table 11. Body mass index^1^ for temporal groups**

a) Summary statistics of body mass index by coarse temporal group.

| **Coarse temporal group** | **n** | **Mean** | **Median** | **SD** | **Range** | **CV** |
| --- | --- | --- | --- | --- | --- | --- |
| Holocene | 438 | 24.08 | 24.52 | 2.00 | 11.08 | 8.31 |
| Late Pleistocene | 59 | 24.80 | 24.45 | 3.30 | 16.49 | 13.31 |
| Middle Pleistocene | 29 | 24.16 | 23.77 | 2.79 | 12.24 | 11.54 |
| Early Pleistocene | 56 | 22.08 | 22.84 | 3.86 | 22.04 | 17.48 |
| Late Pliocene | 3 | 20.80 | 21.92 | 3.48 | 6.70 | 16.73 |

b) Summary statistics of body mass-stature ratios by fine temporal group.

| **Fine temporal group** | **n** | **Mean** | **Median** | **SD** | **Range** | **CV** |
| --- | --- | --- | --- | --- | --- | --- |
| Holocene | 438 | 24.08 | 24.53 | 2.00 | 11.08 | 8.31 |
| Late Pleistocene | 59 | 24.80 | 24.45 | 3.30 | 16.49 | 13.31 |
| late Middle Pleistocene | 6 | 23.77 | 23.22 | 2.61 | 6.88 | 10.98 |
| middle Middle Pleistocene | 17 | 24.81 | 24.89 | 3.11 | 12.24 | 12.43 |
| early Middle Pleistocene | 6 | 22.67 | 22.96 | 1.21 | 3.31 | 5.34 |
| late Early Pleistocene | 3 | 21.26 | 21.15 | 3.65 | 7.30 | 17.17 |
| middle Early Pleistocene | 47 | 22.38 | 22.90 | 4.03 | 22.04 | 18.01 |
| early Early Pleistocene | 6 | 20.06 | 19.66 | 1.84 | 5.05 | 9.17 |
| Late Pliocene | 3 | 20.80 | 21.92 | 3.49 | 6.70 | 16.78 |

^1^ BMI=mass/stature².

**Supplementary Table 12. Body mass index for taxonomic groups.**

a) Summary statistics of body mass-stature ratios by broad taxonomic group.

| **Taxonomic grouping** | **n** | **Mean** | **Median** | **SD** | **Range** | **CV** |
| --- | --- | --- | --- | --- | --- | --- |
| Holocene foragers^a^ | 438 | 24.08 | 24.53 | 2.00 | 11.08 | 8.31 |
| Pleistocene *Homo sapiens^b^* | 42 | 23.57 | 23.36 | 2.70 | 14.05 | 11.46 |
| Neanderthals | 20 | 26.91 | 26.54 | 3.12 | 10.61 | 11.59 |
| SH hominins | 15 | 25.31 | 24.94 | 2.60 | 8.36 | 10.27 |
| Early Mid-Pleistocene *Homo* | 11 | 22.20 | 22.90 | 2.94 | 9.88 | 13.24 |
| Early *Homo* | 39 | 22.78 | 23.19 | 3.29 | 12.12 | 14.44 |
| Australopithecines | 7 | 19.69 | 19.10 | 2.21 | 6.70 | 11.22 |
| Paranthropines | 9 | 20.72 | 18.82 | 6.19 | 22.04 | 29.87 |
| Ardipithecus | 1 | 21.92 | 21.92 | - | - |  |

b) Summary statistics of body mass-stature ratios by narrow taxonomic group.

| **Taxonomic grouping** |  | **n** | **Mean** | **Median** | **SD** | **Range** | **CV** |
| --- | --- | --- | --- | --- | --- | --- | --- |
| Holocene foragers |  | 438 | 24.08 | 24.53 | 2.00 | 11.08 | 8.31 |
| UP modern humans |  | 33 | 24.25 | 23.92 | 2.31 | 10.03 | 9.53 |
| MP *Homo sapiens* |  | 9 | 21.07 | 21.80 | 2.69 | 7.81 | 12.77 |
| *Homo neanderthalensis* |  | 20 | 26.91 | 26.54 | 3.12 | 10.61 | 11.59 |
| Mid-Pleistocene *Homo* |  | 16 | 25.29 | 24.92 | 2.52 | 8.36 | 9.96 |
| *Homo erectus sl* |  | 19 | 23.41 | 23.19 | 3.18 | 10.98 | 13.58 |
| Early *Homo* |  | 22 | 21.98 | 22.84 | 3.22 | 12.00 | 14.65 |
| *Homo habilis* |  | 7 | 22.25 | 23.61 | 3.48 | 8.51 | 15.64 |
| *P. boisei* |  | 6 | 20.71 | 18.33 | 7.62 | 22.04 | 36.79 |
| *P. robustus* |  | 3 | 20.75 | 19.40 | 2.87 | 5.24 | 13.83 |
| *Au. africanus* |  | 5 | 19.47 | 19.10 | 1.24 | 3.18 | 6.37 |
| *Au. afarensis* |  | 2 | 20.23 | 20.23 | 4.74 | 6.70 | 23.43 |
| *Ardipithecus* |  | 1 | 21.92 | 21.92 | - | - |  |

**Supplementary references**

- Aranburu, A., Arsuaga, J. L., & Sala, N. (2107) The stratigraphy of the Sima de los Huesos (Atapuerca, Spain) and implications for the origin of the fossil hominin accumulation. *Quat. Int.* **433**, 5-21.
- Arsuaga, J. L., *et al.* Neandertal roots: Cranial and chronological evidence from Sima de los Huesos. *Science* **344,** 1358–1363 (2014).
- Arsuaga, J. L. *et al.* Postcranial morphology of the middle Pleistocene humans from Sima de los Huesos, Spain. *Proc. Natl. Acad. Sci. USA* **112,** 11524-11529 (2015).
- Bermúdez de Castro, J. M. et al. The Atapuerca sites and their contribution to the knowledge of human evolution in Europe. *Evol. Anthropol.* **13,** 25-41 (2004a).
- Bermúdez de Castro, J.M., Martinón-Torres, M., Lozano, M., Sarmiento, S., Muela, A. Palaeodemography of the Atapuerca-Sima de los Huesos Middle Pleistocene hominid sample. A revision and new approaches to the paleodemography of the European Middle Pleistocene population. *J. Anthropol. Res.* **60**, 5-26 (2004b).
- Carretero, J-M. *et al.* Stature estimation from complete long bones in the Middle Pleistocene humans from the Sima de los Huesos, Sierra de Atapuerca (Spain). *J. Hum. Evol.* **62,** 242–255 (2012).
- Grabowski, M., Hatala, K. G., Jungers, W. L. & Richmond, B. G. Body mass estimates of hominin fossils and the evolution of human body size. *J. Hum. Evol.* **85,** 75-93 (2015).
- Lordkipanidze, D. *et al.* Postcranial evidence from early Homo from Dmanisi, Georgia. *Nature* **449,** 305-310 (2007).
- McHenry, H. M. Femoral lengths and stature in Plio-Pleistocene hominids. *Am. J. Phys. Anthropol.* **85,** 149–158 (1991).
- McHenry, H. M. Body size and proportions in early hominids. *Am. J. Phys. Anthropol*. **87,** 407–431 (1992).
- Mounier, A., Marchal, F. & Condemi S. 2009. Is *Homo heidelbergensis* a distinct species? New insight on the Mauer mandible. *J. Hum. Evol.* **56,** 219–246.
- Pablos, A.*et al*. Human talus bones from the Middle Pleistocene site of Sima de los Huesos (Sierra de Atapuerca, Burgos, Spain). *J. Hum. Evol.* **65**, 79–92 (2013).
- Pablos, A., *et al.* Human calcanei from the Middle Pleistocene site of Sima de los Huesos (Sierra de Atapuerca, Burgos, Spain). *J. Hum. Evol*. **76,** 63-76 (2014).
- Ruff, C. B. & Walker, A. in *The Nariokotome* Homo erectus *Skeleton* (eds Walker, A. & Leakey, R.) 234–265 (Harvard University Press, 1993).
- Stringer C. The status of *Homo heidelbergensis* (Schoetensack 1908). *Evol. Anthropol.* **21,** 101-107 (2012).
- Will, M. & Stock, J. T. Spatial and temporal variation of body size among early *Homo*. *J. Hum. Evol.* **82,** 15-33 (2015).
